# Epigenetics in Pancreatic Ductal Adenocarcinoma: Impact on Biology and Utilization in Diagnostics and Treatment

**DOI:** 10.3390/cancers14235926

**Published:** 2022-11-30

**Authors:** Asmaa Elrakaybi, Dietrich A. Ruess, Michael Lübbert, Michael Quante, Heiko Becker

**Affiliations:** 1 Department of Hematology, Oncology and Stem Cell Transplantation, Medical Center University of Freiburg, Faculty of Medicine, University of Freiburg, 79106 Freiburg, Germany; 2 Department of Clinical Pharmacy, Ain Shams University, Cairo 11566, Egypt; 3 Department of General and Visceral Surgery, Center of Surgery, Medical Center University of Freiburg, 79106 Freiburg, Germany; 4 German Cancer Consortium (DKTK) and German Cancer Research Center (DKFZ), Partner Site Freiburg, 79106 Freiburg, Germany; 5 Department of Gastroenterology and Hepatology, Medical Center University of Freiburg, Faculty of Medicine, University of Freiburg, 79106 Freiburg, Germany

**Keywords:** pancreatic ductal adenocarcinoma, epigenetics, cfDNA methylation, DNMT inhibitors, HDAC inhibitors, retinoids, BET inhibitors, EZH2 inhibitors

## Abstract

**Simple Summary:**

Epigenetic alterations contribute to the distinct biology of pancreatic ductal adenocarcinoma (PDAC) and thus allow a better understanding of molecular mechanisms active in progression, metastasis and therapeutic resistance. Exploiting such knowledge for the development and instalment of clinically impactful biomarkers and epigenetically targeted therapies will open novel and improved avenues for personalized patient care. In this review, we aim to summarize the recent advances in PDAC biology, biomarker development and therapeutic options from an epigenetic perspective.

**Abstract:**

Pancreatic ductal adenocarcinoma (PDAC) is one of the most aggressive malignancies with high potential of metastases and therapeutic resistance. Although genetic mutations drive PDAC initiation, they alone do not explain its aggressive nature. Epigenetic mechanisms, including aberrant DNA methylation and histone modifications, significantly contribute to inter- and intratumoral heterogeneity, disease progression and metastasis. Thus, increased understanding of the epigenetic landscape in PDAC could offer new potential biomarkers and tailored therapeutic approaches. In this review, we shed light on the role of epigenetic modifications in PDAC biology and on the potential clinical applications of epigenetic biomarkers in liquid biopsy. In addition, we provide an overview of clinical trials assessing epigenetically targeted treatments alone or in combination with other anticancer therapies to improve outcomes of patients with PDAC.

## 1. Background

Pancreatic ductal adenocarcinoma (PDAC) is one of the most aggressive solid tumors with a 5-year survival rate of 11% in the United States, making it one of the leading causes of cancer-related mortality [1]. This dismal prognosis is due to several disease- and patient-related factors, such as the diagnosis at advanced stages, tumor localization, age, patient performance status and comorbidities [2,3]. More than 80–90% of patients are diagnosed with irresectable or metastasized disease or develop relapse or metastases after resection, thus require palliative treatment [4]. Depending on the performance status, combination therapies of 5-fluoruracil, irinotecan and oxaliplatin (FOLFIRINOX) or of gemcitabine and nab-paclitaxel or a monotherapy with gemcitabine are the current first-line standard chemotherapy protocols for PDAC patients in the palliative setting [5,6]. However, despite advances, these treatments show modest improvement in overall survival (OS), and can pose a high risk of toxicity.

Early studies have demonstrated the association of initial PDAC histological changes with driver mutations involving, amongst others, the activation of oncogenic KRAS [7] or inactivation of tumor suppressor TP53 [8], SMAD4 [9] or CDKN2A [10], while PDAC progression may rather be related to epigenetic changes [11,12]. In fact, genetic, environmental, and tumor-intrinsic factors, such as the tumor microenvironment (TME), likely collaborate to establish distinct epigenetic landscapes, which shape PDAC heterogeneity [11]. Moreover, uniformity in driver gene mutations between primary tumor and metastatic sites in PDAC patients [13,14] highlight the fact that epigenetic reprogramming is probably a major determinant of clonal fitness and tumor evolution required for PDAC expansion and metastatic spread.

**Figure 1:**
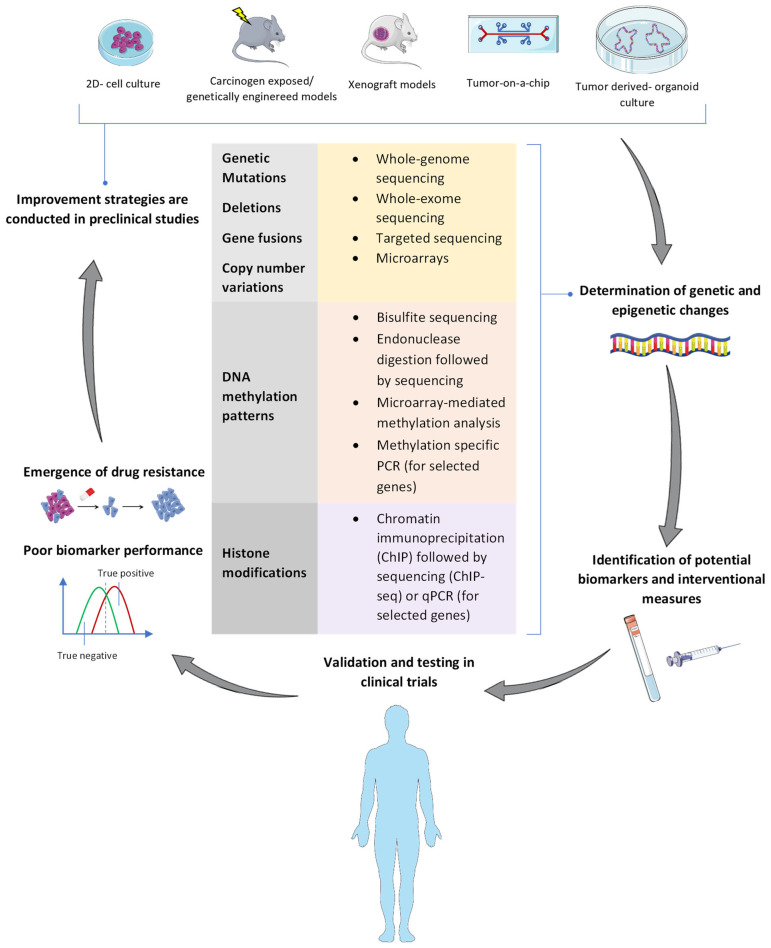
Development of new biomarkers and therapeutic approaches for cancer treatment: A bi-directional process. Bench to bedside; Experimental models used in cancer research can vary from 2D-cell culture to murine in vivo models to more complex 3D patient-derived cancer organoids. These models can identify cancer-related genetic and epigenetic signatures using a plethora of sequencing and targeted qPCR techniques, which can then be utilized to predict novel cancer biomarkers and therapeutic targets to be eventually translated into clinical practice. Bedside to bench; the poor performance of some biomarkers or the emergence of drug resistance to anticancer agents may contribute to their failure to reach the clinic. This urges preclinical studies to test new biomarker panels or to find new strategies to overcome drug resistance with the aim to improve therapeutic outcomes of cancer patients.

The term “epigenetics” was first proposed in the 1940s to describe the mechanism by which a specific genotype could generate different phenotypic effects [15]. In other words, epigenetics bring about divergent gene expression profiles, without altering DNA sequence, but by modulating accessibility of transcription machinery to target genes, a process which is essential to develop cellular identity [16]. Alterations in this mechanism can contribute to tumor evolution by increased cancer cell proliferation and metastasis via silencing tumor suppressor genes or activating oncogenes [17]. Aberrant DNA methylation and post-translational histone modifications are among the main epigenetic alterations, also contributing to PDAC heterogeneity and progression [17]. PDAC harboring mutations in chromatin modifiers (e.g., *ARID1A*, *KMT2C*, *KMT2D*) are more likely to develop a more aggressive squamoid/squamous morphology and metastasis [18]. Moreover, genome-wide analysis of PDAC samples linked the evolution of malignant traits contributing to distant metastasis to widespread epigenetic changes involving global reprogramming of histone H3K9 and DNA methylation within large heterochromatin domains [19]. In that light, ongoing efforts are aiming to develop diagnostic and therapeutic modalities for PDAC based on the dysregulated epigenetic state of the tumor. This should ideally be deployed through a two-way evidence exchange process between preclinical models of varying complexities and data from clinical trials (Figure 1).

## 2. Epigenetic Modifications in the Pathophysiology of PDAC

### 2.1. DNA Methylation

DNA methylation describes the addition of a methyl group to the DNA. Changes in global DNA methylation and of local patterns are among the earliest and most frequent events in cancer development [20]. 5-Methylcytosine (5mC) is the most abundant and best-studied nucleotide modification in eukaryotes. It is generated through the addition of a methyl group to the 5′ carbon of the cytosine pyrimidine ring and predominantly occurs at CpG dinucleotides. Of particular relevance is the cytosine methylation status in the approximately 30,000 CpG islands of the human genome, which are clusters of CpGs located in the gene promoter regions or gene bodies (frequently serving as alternative promoters), the hypermethylation of which typically leads to transcriptional gene silencing [21,22]. Repression of gene expression is facilitated via inhibition of transcription factor binding to the DNA and via chromatin remodeling through the binding of methyl-CpG-binding domain proteins (MBDs) and subsequent recruitment of additional proteins [23,24]. DNA methylation is facilitated by DNA methyltransferases (DNMT) [25]. DNMT3A and DNMT3B establish de novo methylation and DNMT1 maintains methylation in daughter DNA strands. 5mC can be actively demethylated via oxidation to 5-hydroxymethylcytosine (5hmC) by Ten-eleven translocation (TET) dioxygenases which requires α-ketoglutarate (α-KG), which in turn is provided by isocitrate dehydrogenases (IDH) [26].

DNA methylation likely plays a key role in PDAC progression. DNA methylation patterns (globally and at specific loci) differ between PDAC and normal tissue and among PDAC subtypes [27,28,29]. For example, high promoter methylation of the putative tumor suppressor ISL2 in PDAC correlates with poor patient survival and its depletion in human PDAC cells leads to increased oxidative phosphorylation as source for cell energy [30]. Using bisulfite sequencing and methylation-specific PCR (MSP) in PDAC primary tumors and cell lines, DNA hypomethylation and subsequent overexpression of genes altered during tumorigenesis (such as PSCA and S100A4) have been shown to contribute to tumor progression [31]. DNA methylation profiling can distinguish between distinct PDAC subtypes [32]. The more aggressive squamous-/basal-like tumors features hypomethylation of repetitive elements and execution of an intrinsic IFN signaling program that is associated with worse overall survival, compared to the progenitor-like/classical subtype. Moreover, 5hmC has been linked with transcriptional programs defining PDAC subtypes [33]. Loss of 5hmC due to reduced TET2 expression can result in squamous-like PDAC, and enhancing TET2 stability restores 5hmC and GATA6 levels and reverts the phenotype to the classical subtype that features more favorable treatment responses.

Altered DNA methylation may also be a key player in regulating tumor-associated macrophages (TAMs), a main component of the desmoplastic TME in PDAC [34,35,36]. Studies in macrophage cell lines have linked DNMT1-mediated suppression of *SOCS1* expression or of *KLF4* expression with macrophage M1 activation [34,35]. In PDAC-specific models, PDAC cells were able to reprogram M1-like macrophages by inducing DNA methylation which leads to a suppressed glucose metabolic status and a switch of M1-like to M2-like macrophages [36]. In accordance, M1-like macrophages (but not M2-like macrophages) required DNA methylation to promote metastasis in a PDAC mouse model. Moreover, direct contact of PDAC cells with cancer-associated fibroblasts (CAF), another essential component of the TME, induced *SOCS1* methylation with downstream activation of STAT3 and insulin-like growth factor (IGF)-1 expression [37]. These results are in line with PDAC cells being in constant interaction with the TME to support their growth, progression and metastasis formation.

Changes in DNA methylation patterns strongly correlate with aging [38,39], with “epigenetic clocks”, i.e., the methylation status of a set of CpG sites, being able to reliably predict an individual’s age. The methylation status is under the influence of extrinsic factors (e.g., nutrition, microbiome) and of a process called “epigenetic drift” [38]. In this process, errors occur in the transfer of epigenetic marks to the daughter DNA strands due to the relatively low fidelity of DNMTs. PDAC is usually referred to as a disease of the elderly [40], with less than 10% of the patients being younger than 55 years [41]. This raises the question whether DNA methylation patterns may be associated with PDAC development in younger patients. However, a study by Raffenne and colleagues using publicly available DNA methylation data found no difference in the DNA methylation profiles between early- and late-onset PDAC [42]. In another study, DNA methylation (as a sign of aging) in leukocytes were found to be associated with an increased risk for PDAC [43]. Given for example the potential for identifying younger individuals at higher risk for cancer development (including PDAC) through age-associated DNA methylation and other epigenetic marks, extended research in this context appears warranted.

### 2.2. Histone Modifications

In eukaryotic cells, nucleosomes are the basic structural unit of DNA packaging, where DNA is wrapped around histone octamers allowing its condensation to chromatin [44]. N-terminal histone tails protruding from nucleosomes are prone to posttranslational modifications. Acetylation and deacetylation of lysine residues in these histone tails, mediated by histone acetyltransferases (HATs) and histone deacetylases (HDACs), are important mechanisms to regulate chromatin accessibility and gene transcription [16]. Enhanced acetylation is associated with a more relaxed chromatin accessible to the transcription machinery, while the reverse reaction facilitates gene silencing. While the function of HATs (e.g., p300) in PDAC could be either tumor suppressing or promoting depending on the targeted genes [45,46], the role of HDACs seems to be more consistent. HDACs are able to mediate tumorigenesis, and their activity is associated with poor outcomes in PDAC patients [47,48], for example owing to the suppression of genes encoding proapoptotic proteins such as BH3-only protein NOXA and Nur77 with subsequent enhancement of cellular proliferation [48].

Acetylated lysine residues are recognized by proteins of the bromodomain and extra-terminal (BET) family (including BRD2, BRD3, BRD4, BRDT) [49]. Binding to hyperacetylated chromatin regions leads to formation of a super enhancer protein complex and interaction with the positive transcription elongation factor (P-TEFb) which promotes gene transcription and elongation [50,51]. BET protein dysregulation can for example be involved in tumor development and progression by promoting the expression of classical oncogenes such as *MYC* [52].

Lysine residues in the histone tails can also serve as methylation targets for histone methyltransferases (HMTs), while these marks can be removed by histone demethylases (HDMs) [16]. The effect of histone methylation on gene expression is context-dependent and relates to the lysine position [53]. For instance, trimethylation of lysine 4 in histone 3 (H3K4me3) is generally associated with gene activation, while the contrary occurs with trimethylation of lysine 27 (H3K27me3). Aberrant histone methylation of cancer-related genes has been involved in abnormal proliferation, cell cycle dysregulation, immune escape and metabolic reprogramming of tumor cells [54]. ChIP-seq data demonstrated that gain of H3K27me3 and loss of H3K4me3 at acinar cell fate genes enhanced acinar-to-ductal metaplasia which is essential for PDAC development and progression [55]. Loss of KDM6A, an HDM of H3K27me3, in a PDAC mouse model induced aggressive squamous-like, metastatic disease related to the activation of H3K27ac-marked enhancers regulating *ΔNp63*, *MYC* and *RUNX3* [56]. The HMT enhancer-of-zeste homolog 2 (EZH2), the catalytic component of the polycomb repressive complex 2 (PRC2), mediates generation of H3K27me3 [57]. EZH2 was found to be overexpressed in the nucleus in PDAC cell lines and in 68% of PDAC cases, and depletion of EZH2 decreases PDAC cell proliferation [58] and induces a less aggressive and more chemotherapy-susceptible, classical PDAC subtype (likely via increased *GATA6* expression) [59].

### 2.3. Epigenetic Characteristics of Metastatic PDAC

PDAC is characterized by high frequency of metastases [60] with common sites of dissemination including liver, peritoneum and lungs [61]. Epithelial to mesenchymal transition (EMT) is considered a major regulator of tumor spread, where cancer cells lose their epithelial markers such as E-cadherin, while gaining mesenchymal and fibroblast-like properties [62]. Clinical and preclinical studies showed an inverse correlation between EZH2 expression and E-cadherin in PDAC [63], where high EZH2 expression was associated with advanced disease stage and lymph node metastasis [64]. Moreover, expression of *CDH1*, encoding E-cadherin, is downregulated in pancreatic cancer cells by binding of repressor complexes comprised of HDACs and certain transcription factors such as ZEB1 or Snail [65,66].

FOXA1 and FOXA2 are transcription factors which induce the expression of E-cadherin [67]. Consequently, their downregulation was associated with EMT induction and cancer progression in in vivo and in vitro PDAC models. It was also implicated, by results of ChIP-seq, RNA-seq and ATAC-seq, that FOXA1 enhanced H3K27ac in certain genomic regions in PDAC cells, which activated foregut developmental genes, thus promoting cellular growth in vitro and metastasis in vivo [68]. Further studies are required to explain these observations.

Aberrant DNA methylation has also been implicated in PDAC metastasis [69,70,71]. TFPI-2, a proteinase inhibitor which prevents extracellular matrix degradation and thereby tumor invasion and metastasis, is downregulated in PDAC tumors and cell lines owing to its hypermethylated promoter as revealed by MSP and bisulfite sequencing [69]. Restoration of its expression reduced the malignant behavior of PDAC in vitro. Similar effects were observed for the promoter methylation of the *RELN* gene, which encodes an extracellular matrix serine protease regulating neuronal migration and the low expression of which associated with worse survival in pancreatic cancer [70]. In addition, DNA hypomethylation of *MET* (encoding c-Met) and *ITGA2* (encoding Integrin α-2) correlated with high gene expression and with poor patient outcomes [71].

Hence, epigenetic modifications (e.g., chromatin remodeling or altered DNA methylation) can initiate transcriptional changes in PDAC and thus promote the gain of aggressive and metastatic disease characteristics.

## 3. Diagnostic Utility of Epigenetic Modifications in PDAC

### 3.1. DNA Methylation in Liquid Biopsies as Marker for the Diagnosis of PDAC

As discussed earlier, most PDAC cases are diagnosed at advanced stages which is related to the absence of specific signs and symptoms during the early phases of PDAC and the tendency to early spread [72]. Due to this delay in detection, less than 20% of patients qualify for primary surgical resection [73]. The standard tumor biomarker at PDAC diagnosis is carbohydrate antigen 19-9 (CA 19-9) [74]. However, owing to its low sensitivity and specificity, its application for early PDAC screening is not recommended. Biomarkers which provide a better performance for early diagnosis of PDAC are required.

Liquid biopsy refers to the detection of cancer cells or cell material in blood and other body fluids [75]. Liquid biopsy approaches are currently usually based on the analysis of plasma cell free DNA (cfDNA). Most of the (particularly initial) liquid biopsy studies in PDAC focused on the detection of gene variants, especially *KRAS* mutations [76,77,78,79,80]. However, DNA methylation marks in cfDNA of PDAC patients have also been studied, and they may add clinically relevant information, in particular in combination with genetic analyses.

Melnikov et al. were among the first to study methylation changes of cfDNA in PDAC [81]. They were able to determine a classifier based on the promoter methylation of five genes that differentiated patients with PDAC from healthy controls, but sensitivity (76%) and specificity (59%) were still modest. The ability of cfDNA methylation to identify patients with PDAC has since then been investigated in numerous studies [82,83,84,85,86,87,88,89,90]. In another early study comprising 104 patients with PDAC and assessing *NPTX2* hypermethylation in cfDNA, sensitivity and specificity were 80% and 76% to identify patients with PDAC [83]. The hypermethylation of *NPTX2*, together with that of *SPARC*, in cfDNA also correlated with PDAC diagnosis (vs. chronic pancreatitis) and with poor survival in another study [84]. The promoter methylation of *BNC1* and *ADAMTS1* were also identified as promising cfDNA markers for the detection of PDAC [86]. This was recently corroborated by the observation that the combined assessment of these markers achieved a sensitivity of 97.4% and specificity of 91.6% to distinguish patients with PDAC from controls [85]. Henriksen and colleagues analyzed a 28-gene panel and defined a prediction model comprising higher age and methylation status of 8 genes (*BMP3*, *RASSF1A*, *BNC1*, *MESTv2*, *TFPI2*, *APC*, *SFRP1*, *SFRP2*) to differentiate between PDAC patients and those with pancreatitis or no pancreatic disease with a sensitivity of 76% and specificity of 83% [87]. The concurrent analyses of hundreds of methylation marks in cfDNA also allowed for the differentiation among various gastrointestinal cancers, including PDAC [89]. In a recent study, a set of 10 cfDNA methylation markers (*MIR129-2*, *LINC01158*, *CCDC181*, *PRKCB*, *TBR1*, *ZNF781*, *MARCH11*, *VWC2*, *SLC9A3*, *HOXA7*) demonstrated a very good performance with 100% sensitivity at 95% specificity to distinguish between metastatic pancreatic cancer and benign pancreatic cysts [91].

Adding another diagnostic modality (CA19-9 levels, *KRAS* mutation status etc.) to the assessment of cfDNA methylation can improve accuracy [90,92,93]. Evaluating CA 19-9 levels together with the methylation status of *RUNX3* in cfDNA was able to increase sensitivity to detect PDAC from 50.9% (*RUNX3* DNA methylation alone) to 85.5% [92]. In another study, cfDNA analyses of 13 methylation markers among 120 advanced-stage and 50 early-stage PDAC patients and 170 controls showed that the combined analyses of the DNA methylation markers and CA19-9 levels compared significantly better with either assays alone, with an overall sensitivity and specificity of 92% at the pre-set specificity of 97.5% [90].

DNA methylation markers have also been investigated in body fluids other than plasma or serum [94,95,96]. In one study, 14 markers were studied in pancreatic juice samples from 38 patients with PDAC or intraductal papillary mucinous neoplasms (IPMN) with high grade dysplasia and were compared with controls (*N* = 73) [94]. A group of 3 markers (*C13orf18*, *FER1L4*, *BMP3*) was sufficient to distinguish patients with pancreatic cancer from controls with 83% sensitivity at a pre-set specificity of 86%. The same group analyzed a set of 13 methylation markers in 134 pancreatic cyst fluid samples, including 21 cases with PDAC or high grade dysplasia and 113 controls [95]. Two markers (*TBX15*, *BMP3*) achieved a sensitivity and specificity of > 90%. The group had previously also assessed DNA testing (methylation markers and *KRAS* mutations) from stool for the detection of PDAC [96]. At 90% specificity, the combination of methylated *BMP3* and mutant *KRAS* detected 67% of PDAC patients.

### 3.2. DNA Methylation in Liquid Biopsies as Marker for Prognostication and Treatment Monitoring of PDAC

The importance of analyzing cfDNA methylation cannot only be limited to PDAC diagnosis, it may become of clinical significance for prognostication of the disease and treatment monitoring [91,97,98,99,100].

In one study, the mean number of hypermethylated genes in cfDNA was significantly higher in metastatic (that means prognostically unfavorable) disease than in earlier stages of PDAC [97]. The same group showed that patients with more than 10 hypermethylated genes of a 28 gene panel had worse survival outcomes than those with fewer [98].

In addition to the mere number of hypermethylated genes, the specific set of aberrantly methylated genes in cfDNA can have prognostic potential [97,98,99,100]. For example, hypermethylation of *ALX4*, *BNC1*, *HIC1*, *SEPT9v2*, *SST*, *TFPI2*, and *TAC1* differed between stage IV and stage I-III disease in the aforementioned study [97]. Based on the gene methylation status, there have been attempts to establish prognostic models but they require further validation [98,99]. Of interest is a post hoc analyses of the Prodige 35 and Prodige 37 trials, in which cfDNA was assessed for two methylation markers (*HOXD8*, *POU4F1*) in 354 patients [100]. Median progression-free survival (PFS) and OS were 5.3 and 8.2 months in cfDNA-positive and 6.2 and 12.6 months in cfDNA-negative patients, respectively. In multivariable analyses, the cfDNA methylation status remained an independent prognosticator for PFS (hazard ratio (HR) 1.5) and OS (HR 1.62).

Owing to its non-invasive nature, plasma cfDNA allows serial monitoring of tumor burden and evolution under treatment, which cannot be realized by tissue biopsy [101]. Although data on cfDNA methylation under treatment are scarce, a decrease in cfDNA methylation levels has been reported in patients undergoing chemotherapy [91].

In summary, assessment of cfDNA methylation has promising diagnostic and prognostic value in PDAC. Further validation studies in larger patient cohorts are required to determine the most suitable DNA methylation biomarker panel for early detection, prognostication and monitoring of PDAC patients.

### 3.3. Histone Modifications in Liquid Biopsies as Biomarker in PDAC

As mentioned earlier, nucleosomes are complexes of DNA and histone proteins which constitute chromatin [44]. In several conditions, including cancer, mono- and oligonucleosomal fragments are released during cellular apoptosis into the blood circulation, where they can potentially be used for diagnostic purposes [102]. One study showed that markers of epigenetic modifications (e.g., histone modifications, of circulating nucleosomes were able to distinguish between PDAC patients and control cases with good performance (72% sensitivity at pre-set 90% specificity) [103]. In the same study, consideration of CA 19-9 in addition to a panel of 4 epigenetic markers enhanced the sensitivity to 92%. However, further research is needed to confirm these findings.

### 3.4. Liquid Biopsy to Select Epigenetically Active Treatment in PDAC

As mentioned, the analyses of certain gene mutations in cfDNA may add to the diagnostic and prognostic value of epigenetic biomarkers in PDAC. In that light, *KRAS* mutation status in PDAC patients may inform on the sensitivity of decitabine, a DNMT inhibitor, which exerted its anti-tumor effects in *KRAS*-mutated PDAC [104]. In fact, a current phase II study is aiming to provide a proof-of-concept that KRAS-dependent PDACs are responsive to decitabine treatment [105]. Similarly, mutations or loss of components of the SWI/SNF (SWItch/Sucrose Non-Fermentable) complex, which is involved in the spatial organization of chromatin, might become of relevance in PDAC [106]. In a phase I study, solid tumors bearing loss of SWI/SNF subunit expression showed increased sensitivity to the EZH2 inhibitor tazemetostat [107], which was consistent with previous preclinical findings showing oncogenic dependency of SWI/SNF mutated cells on EZH2 activity [108]. Thus, SWI/SNF status-guided treatment with EZH2 inhibitors may become a promising approach in PDAC treatment.

Table 1 summarizes the hitherto mentioned studies assessing the diagnostic and prognostic value of liquid biopsy testing of epigenetic biomarkers in PDAC.

## 4. Epigenetic-Based Therapeutic Approaches

As summarized above, epigenetic modifications play a key role in PDAC development and in tumor-to-metastasis transition. It is hence not surprising to find that treatment strategies based on targeting epigenetic regulators recently became a subject of research interest in PDAC, as outlined in the following section. The clinical trials discussed in this part are summarized in Table 2.

### 4.1. DNMT Inhibitors (DNMTi)

While DNA hyper- and hypomethylation are both implicated in cancer development, hypermethylation of tumor suppressor genes and DNMT overexpression are established as major players in carcinogenesis [109]. The DNMTi azacitidine (5-azacytidine) and its deoxy-derivative decitabine (5-aza-2′-deoxycytidine) are cytidine analogues that are incorporated into DNA upon replication [109]. This leads to irreversible binding of DNMT1 resulting in its degradation and decreased DNA methylation. Azacitidine, in contrast to decitabine, is additionally and mostly incorporated into RNA which inhibits polyribosome assembly and protein generation. As single agents, DNMTi are currently approved for treatment of myelodysplastic syndromes (MDS) or acute myeloid leukemia (AML) [109]; in AML, also in combination with the BCL2 inhibitor venetoclax [110]. DNMTi demonstrated particular clinical benefit in patients with MDS or AML with adverse genetics, such as *TP53* aberrations [111].

When treated with azacitidine, the PDAC cell line PANC-1 showed less tumorigenicity, which was associated with re-expression of antiproliferative somatostatin (SST) and its receptor 2 (SSTR2) [112]. Growth inhibition was further increased after the addition of gemcitabine. In line with these findings, a phase Ib clinical trial has been conducted to test the safety and efficacy of decitabine plus gemcitabine in patients with advanced PDAC and sarcoma [113].

Additionally, in PDAC cells isolated from a stroma-rich mouse model (KPC-Brca1 mice) IFN-inducible genes (including *STAT1* and *STAT2*) were overexpressed upon decitabine treatment, and combination of IFN-γ with decitabine demonstrated an additive antiproliferative effect on PDAC cells [114]. Azacitidine was shown to enhance tumor T-cell infiltration and expression of transcripts for antigen presentation machinery such as MHC class I in mouse and human PDAC cell lines, which was associated with tumor regression in azacitidine treated mice [115,116]. Therefore, sensitization to immune checkpoint therapy by DNMTi has been subject to several phase I/II trials in patients with advanced PDAC [117,118,119] (Table 2).

Systemic elevation of cytidine deaminase (CDA) levels, which rapidly metabolizes cytidine analogues into inactive uridine, is a potential resistance mechanism to decitabine [120]. Accordingly, combining DNMTi with high doses of CDA inhibitors is considered a promising treatment strategy to overcome resistance in patients with advanced PDAC, although currently available clinical data have been unsatisfactory [121,122]. Further ongoing and completed phase I/II studies of DNMTi in different PDAC patients are illustrated in Table 2 [123,124].

### 4.2. HDAC Inhibitors (HDACi)

HDACi can modulate expression of genes involved in apoptosis, differentiation and angiogenesis and inhibit PDAC tumor growth by restoring the histone acetylation balance [125].

In pancreatic cell lines, HDACi have shown promising antitumor effects as monotherapy [126,127] as well as in combination with other therapeutic agents such as gemcitabine [128] and proteasome inhibitors [129]. Several phase I and II clinical trials investigated safety, and to some extent, efficacy of HDACi monotherapy or in combination with chemotherapeutic agents or proteasome inhibitors in PDAC treatment [130,131,132,133,134,135,136,137,138,139,140,141,142] (Table 2). Nevertheless, the clinical efficacy of the additional HDACi application remained unsatisfactory in most studies, while being associated with hematologic and gastrointestinal toxicities.

Similar to DNMTi, the immunomodulating effects of HDACi have increasingly moved into the focus [143,144,145]. HDACi restores MHC I surface expression in tumor cells deficient of TAP, a component of the antigen processing machinery, and enhances immunogenicity and T-cell infiltration [143]. In a metastatic PDAC mouse model, HDACi application reduced the immunosuppressive ability of granulocytic myeloid-derived suppressor cells (G-MDSCs) in the TME, leading to sensitization to immune checkpoint inhibitor treatment [144]. To that end, a current phase II trial is aiming to determine the efficacy of the HDACi entinostat with the PD1 inhibitor nivolumab in patients with unresectable PDAC [145].

Moreover, combining HDACi with other targeted therapies, for example tyrosine kinase inhibitors (TKIs), may be an approach to modify HDACi effects in PDAC, as it has been investigated in a phase II trial, which tested the combination of the HDACi vorinostat and the TKI sorafenib with gemcitabine and radiation therapy [146] (Table 2). This might extend the findings of a recent study in hepatocellular carcinoma, where the HDACi resminostat in combination with sorafenib inhibited platelet-mediated cancer promoting effects, possibly via reduction of platelet-induced CD44 expression, suppression of EMT and MEK/ERK signaling [147]. In fact, combining HDACi with inhibitors of MEK and PI3K, the downstream effectors of KRAS signaling, enhanced apoptosis and reduced metastasis, therapeutic resistance and self-renewal of PDAC cells [148,149], underscoring the potential of KRAS targeting as a promising treatment in combination with HDACi in PDAC patients.

### 4.3. Retinoids

Retinoids are derivatives of vitamin A. The first generation retinoid all-*trans* retinoic acid ATRA (Tretinoin) is approved for treatment of acute promyelocytic leukemia (APL) [150]. ATRA also increases the efficacy of decitabine, without added toxicity, in frail patients with AML other than APL [151]. ATRA treatment induces changes in chromatin conformation/accessibility [152,153] and acts synergistically with decitabine [152] and HDACi [154]. It also has demonstrated (although mostly moderate) single-agent efficacy in various solid tumors [155].

Retinoid signaling is fundamental in normal pancreas and PDAC development [156]. ATRA by itself exerts antineoplastic effects and increases cytotoxic effects of gemcitabine in PDAC [157,158]. ATRA can restore quiescence of fibroblasts (through PIN1 inhibition), which reduces desmoplastic features in the TME of PDACs and thus decreases chemotherapy resistance [159,160,161]. ATRA has been investigated in combination with gemcitabine and nab-paclitaxel in a phase I trial and showed an expectedly excellent toxicity profile and encouraging response rates and duration, which led to a planned randomized phase II trial [162,163,164] (Table 2).

Recently, the combination of the HDACi belinostat with 13-*cis*-retinoic acid (isotretinoin, prodrug of ATRA) was well tolerated in patients with advanced solid tumors, including three with PDAC [165], which might prompt more studies to further assess its efficacy in PDAC. Moreover, retinoids enhanced the response to immune checkpoint inhibition, by inducing interferon mediated inflammation in TME, which was characterized by increased CD8+ T cell and decreased T-reg infiltration in cancer models [166]. On that basis, a phase I study is currently underway to test the efficacy of ATRA and nivolumab combination in patients with advanced or metastatic PDAC [167] (Table 2).

### 4.4. BET Inhibitors (BETi)

BETi, which competitively bind the acetyl-lysine recognition motif at the bromodomain of BET proteins, can repress expression of oncogenes including those of known relevance in PDAC, such as c-MYC [168,169]. BETi displayed significant in vitro and in vivo anti-tumorigenic activity individually and increased the therapeutic effects of other treatment modalities in PDAC [170,171,172,173,174]. The potential benefits of BETi concluded from preclinical studies remain to be confirmed in patients [175,176]. The BETi mivebresib displayed modest efficacy, with 26 of 61 patients with solid tumors (including PDAC) achieving stable disease, while the remaining patients had disease progression [175]. While BETi monotherapy may not be an optimal therapeutic option, its role in combination with other systemic therapies or with radiotherapy requires further assessment. In line with this, the BETi JQ1 and vorinostat synergistically suppressed tumor growth in a mouse model for advanced PDAC [177]. Similar results were obtained in PDAC cell lines and xenograft models with a dual BET/HDAC inhibitor [178]. In another study, JQ1 attenuated DNA double-strand repair and consequently sensitized the tumor cells to PARP inhibitors (PARPi), both of which exerted synergistic cytotoxic activity in vitro and in patient derived xenograft (PDX) models of PDAC [179]. To test the applicability of these approaches in PDAC and other solid tumors, phase I/II studies are currently testing the combination of the HDACi entinostat with the BETi ZEN-3694 [180] and of the BETi NUV-868 with the PARPi olaparib and the antiandrogen enzalutamide [181].

Reprogramming immune response by epigenetic modifications comes into play, when a dual BET/HAT inhibitor enhanced antigen presentation of PANC-1 cells and T cell recruitment to the tumor stroma, and sensitized PDAC cells to immune checkpoint inhibition and extended survival in the KPC mouse model of advanced PDAC [182]. In a study involving PDAC PDX models and patient biopsies, recruitment of TNF-α^+^ macrophages, mediated by BRD4-mediated cJUN/AP1 expression, shifted tumor cells from the classical to the aggressive basal subtype, which was reversed upon treatment with the BETi JQ1 [183]. Current phase I/II trials are examining this treatment approach in advanced solid tumors [184,185].

### 4.5. EZH2 Inhibitors (EZH2i)

The methyl group added by the HMT EZH2 is provided by S-adenosylmethionine (SAM) which is demethylated to S-adenosylhomocysteine (SAH) [186]. The majority of EZH2i (e.g., tazemetostat) competitively occupy the site for SAM in the binding pocket of EZH2. Different from that, 3-deazaneplanocin A (DZNep) inhibits SAH degradation which causes methyl accumulation, which in turn inhibits EZH2 enzyme activity. EZH2i lead to deprivation of the enzymatic activity of EZH2, which for example contributes to low H3K27me3 levels and subsequent anti-tumor effects [186].

Preclinical studies demonstrated the synergistic cytotoxic effects of EZH2i in combination with other treatment modalities in several models of solid tumors [187,188,189,190]. In a panel of PDAC cells, DZNep enhanced the anti-proliferative effects of gemcitabine and reduced cellular migration potentially via augmenting expression of E-cadherin [191]. Dual EZH2 and BET inhibition reduced colony formation, induced cell cycle arrest and caused apoptosis in PDAC cell lines, better than each individual inhibitor alone, and suppressed tumor growth in xenograft mice models [192]. Such data on EZH2i combination therapies need to be considered when seeing the minor efficacy of EZH2i monotherapy, as indicated by GSK2816126 treatment of patients with advanced hematologic or solid malignancies (including PDAC) despite its relative safety [193].

Several ongoing and completed phase I/II studies have been dedicated to further explore the safety and efficacy of EZH2i mono- and combination therapies in hematologic and solid cancers [194,195,196,197,198,199]. Of these, two studies aim to assess the potential benefits of combining tazemetostat with immune checkpoint inhibition [198,199]. This concept is supported by the finding that EZH2i treatment enhanced antigen presentation in head and neck squamous cell carcinoma cells and cytotoxicity of CD4+ and CD8+ T cells, and improved response of anti-CTLA-4 and anti-PD-1 immune checkpoint inhibitors in solid tumor models [200,201].

Most studies exploring the synergism of epigenetically active drugs with other treatment modalities studied combinations with chemo- and/or radiotherapy, targeted therapies, or immunotherapies. Two ongoing trials assess the safety and efficacy of combining epigenetically active drugs, i.e., HDACi with retinoids or with BETi, in patients with solid tumors including PDAC [165,180]. DNMTi/HDACi combinations have not demonstrated convincing added efficacy in several phase I/II trials in hematologic malignancies or solid tumors [151,202]. Considering preclinical studies in PDAC, the anti-tumoral effect of the DNMTi zebularine was augmented, when combined with the HDACi SAHA in PDAC cell lines, which was, however, not reproducible in xenograft models [203]. On the other hand, enhanced tumor suppression was observed when a BETi was combined with HDACi, HATi or EZH2i in PDAC cell lines and mouse models, as discussed earlier in this section, which may provide a promising strategy [177,178,182,192].

**Table 2:** Clinical trials evaluating epigenetic therapy in pancreatic cancer. The list may not be exhaustive, particularly since not yet published data of trials in solid tumors do not allow conclusions on the number of patients enrolled with pancreatic cancer.

Type	Drug/Route of Administration	Combination	Comparison	Phase (Status)	Condition	Pt number and Results	Reference
**DNMTi**	Decitabine iv	-	-	II (r)	PDAC (unresectable or metastatic)	No results reported	NCT05360264 [105]
	Decitabine sc	Gemcitabine	-	I (a)	PDAC (metastatic) Sarcoma	No results for PDAC reported	NCT02959164 [113]
	Decitabine po	Tetrahydrouridine	-	I (c)	PDAC (metastatic)	13 pts; 8 evaluable pts: SD *n* = 1, PD *n* = 7, median OS 3.1 mo	NCT02847000 [121,122]
	Azacitidine sc	Pembrolizumab	-	II (a)	PDAC (unresectable or metastatic)	36 pts; 34 evaluable pts: PR *n* = 3, SD *n* = 8, median OS 4.67 mo 21% ≥ G3 AE	NCT03264404 [117]
	Azacitidine sc	Romidepsin nab-Paclitaxel Gemcitabine Durvalumab Lenalidomide	-	I/II (r)	PDAC (metastatic)	No results reported	NCT04257448 [118]
	Azacitidine po	-	Observation (OBS) (1:1)	II (c)	PDAC (after adjuvant chemotherapy)	48 evaluable pts: PFS HR 1.01, OS HR 1.01, median PFS 7.8 mo (AZA) vs. 8.9 mo (OBS), median OS 21.9 mo (AZA) vs. 25.6 mo (OBS)	NCT01845805 [123]
	Azacitidine po	Carboplatin nab-Paclitaxel	-	I (c)	Solid tumors	PDAC (part 2): 24 evaluable pts: DCR 46%	NCT01478685 [124]
	Guadecitabine iv	Durvalumab	-	I (a)	PDAC HCC BTC	PDAC: 24 evaluable pts: PR *n* = 1, SD *n* = 7, median PFS 2.1 mo, median OS 4.4 mo	NCT03257761 [119]
**HDACi**	Belinostat iv	Carboplatin Paclitaxel	-	I (c)	Solid tumors	PDAC: 3 pts: PR *n* = 1	[130]
	Tacedinaline po	Gemcitabine	Gemcitabine (1:1)	II (c)	PDAC (unresectable or metastatic)	174 evaluable pts: ORR 12% vs. 14%, OS HR 0.98, median OS 6.5 mo vs. 7.1 mo	NCT00004861 [133]
	Vorinostat po	Capecitabine Radiotherapy	-	I (c)	PDAC (resectable, borderline resectable, unresectable)	21 pts: median OS 13.2 mo	NCT00983268 [139]
	Vorinostat po	Marizomib	-	I (c)	PDAC NSCLC Melanoma	PDAC: 2 pts	NCT00667082 [131,140]
	Panobinostat po	Bortezomib	-	II (c)	PDAC (metastatic)	7 evaluable pts: PD *n* = 7, median PFS 0.86 mo, median OS 4.01 mo	NCT01056601 [134]
	Vorinostat po	Bortezomib	-	I (c)	Solid tumors	PDAC: 6 pts	NCT00227513 [135]
	Valproic acid po	S-1	-	I/II (c)	PDAC (unresectable or metastatic) BTC	PDAC: 7 pts	[136]
	Mocetinostat po	Gemcitabine	-	I/II (c)	Solid tumors	PDAC: 13 evaluable pts (ph II): SD *n* = 9, median PFS 5.3 mo, median OS 7.4 mo	NCT00372437 [137]
	Resminostat po	S-1	-	I (c)	PDAC (unresectable or metastatic) BTC	PDAC: 7 pts; 3 evaluable pts (regimen 3): SD *n* = 2, median PFS 2.3 mo, median OS 4.7 mo	[138]
	Vorinostat po	Gemcitabine Sorafenib Radiotherapy	-	I (a)	PDAC (resectable, borderline resectable, unresectable)	22 pts	NCT02349867 [146]
	Romidepsin iv	Gemcitabine	-	I (c)	PDAC (unresectable or metastatic) Other solid tumors	27 evaluable pts; SD *n* = 14, PD *n* = 11; 67% ≥G3 AE	NCT00379639 [132,141]
	Romidepsin iv	-	-	I (a)	Solid tumors Lymphoma	PDAC/BTC: 5 pts	NCT01638533 [142]
	Entinostat po	Nivolumab		II (c)	PDAC (unresectable or metastatic) BTC	PDAC: 18 evaluable pts: CR/PR *n* = 3, median OS 3.9 mo; 63% ≥G3 AE	NCT03250273 [145]
**Retinoids**	ATRA po	Gemcitabine nab-paclitaxel	-	I (c)	PDAC (unresectable or metastatic)	28 pts; 15 evaluable pts: PR *n* = 7, SD *n* = 7, median OS 11.7 mo; 63% ≥G3 AE	NCT03307148 [162,163]
	ATRA po	Gemcitabine nab-paclitaxel	Gemcitabine nab-paclitaxel	II (not yet recruiting)	PDAC (unresectable)	No results reported	NCT04241276 [164]
	Isotretinoin po	Belinostat	-	I (c)	Solid tumors	PDAC: 3 pts: CR/PR/SD *n* = 0	NCT00334789 [165]
	ATRA po	Nivolumab	-	I (a)	PDAC (unresectable or metastatic)	No results reported	NCT05482451 [167]
**BETi**	Mivebresib po	-	-	I (c)	Solid tumors	PDAC: 6 evaluable pts; 56% ≥G3 AE	NCT02391480 [175]
	Birabresib po	-	-	I (c)	Solid tumors	No results for PDAC reported	NCT02259114 [176]
	ZEN-3694 po	Entinostat	-	I/II (r)	Solid tumors Lymphomas	No results reported	NCT05053971 [180]
	NUV-868 po	Olaparib Enzalutamide	-	I/II (r)	Solid tumors	No results reported	NCT05252390 [181]
**EZH2i**	Tazemetostat po	-	-	I (c)	Solid tumors Lymphomas	No results for PDAC reported	NCT01897571 [107]
	GSK2816126 iv	-	-	I (c)	Solid tumors Lymphomas	PDAC: 2 pts	NCT02082977 [193]
	Tazemetostat po	Durvalumab	-	II (r)	Solid tumors	No results reported	NCT04705818 [199]

a, active not recruiting, AE, adverse events; AZA, azacitidine; BETi, bromodomain and extra-terminal proteins inhibitors; BTC, biliary tract cancer; c, completed; CR, complete remission; DCR, disease control rate (CR + PR + SD); DNMTi, DNA methyl transferase inhibitors, EZH2i, enhancer-of-zeste homolog 2 inhibitors; G3, grade 3; GI, gastrointestinal; HCC, hepatocellular carcinoma; HDACi, histone deacetylase inhibitors; iv, intravenous; mo, months; NSCLC, non-small cell lung cancer; OBS, observation; ORR, overall response rate (CR + PR); OS, overall survival; PD, progressive disease; PDAC, pancreatic ductal adenocarcinoma; PFS, progression-free survival; po, per os (oral administration); PR, partial remission; pts, patients; r, recruiting; SD, stable disease.

## 5. Conclusions and Perspective

In addition to genetic aberrations, dysregulation of epigenetic mechanisms including DNA methylation and histone modifications are main contributors to PDAC biology and heterogeneity, and hence, disease progression, metastasis and chemoresistance. Future expansion of recent single-cell RNA sequencing data by integrative single-cell sequencing analyses of genetic and epigenetic aberrations will help to even better define the spatial and intercellular heterogeneity and its changes during tumor evolution and under treatment [204,205,206].

The uniformity in driver gene mutations between primary tumors and metastatic sites but potential differences in biology and treatment response indicate that epigenetic alterations contribute to PDAC metastasis and tumor migration [13,14]. Aberrant DNA methylation and chromatin remodeling are involved in the loss of epithelial cell adherence and gain of mesenchymal-like features, while enhancing extracellular matrix degradation, which promotes PDAC migration, invasiveness and resistance to therapy [63,64,65,66,68,69,70,71].

Utilizing epigenetic information for the development of reliable biomarkers and successful therapeutic strategies is of essence. Liquid biopsy is emerging as a reliable and non-invasive biomarker approach for diagnosis, prognostication and/or treatment monitoring in PDAC. cfDNA methylation patterns are able to differentiate between PDAC and benign pancreatic conditions with already relatively high accuracy [81,82,83,84,85,86,90,91,92,94,95,96]. Moreover, cfDNA methylation markers have demonstrated promising results for identifying metastatic stage and estimating the prognosis of PDAC patients [84,97,98,99,100]. In light of the heterogeneous nature of PDAC, future studies should be dedicated in developing biomarker panels, that combine epigenetic data with other modalities (e.g., CA 19-9 levels or gene mutation status) to improve the prediction performance and aid in developing tailored therapy [90,92,103,105,107]. As illustrated in Figure 1, the development of biomarkers has to be performed hand-in-hand with novel treatment modalities to allow for an optimum of prognostic and predictive information. Only a few studies have described the predictive value of molecular markers in the context of epigenetically active treatment in PDAC, e.g., *KRAS* mutation status for DNMTi [104,105], SWI/SNF status for EZH2i [107,108] or expression status of FABP5 for retinoids [207].

Combining epigenetically targeted therapies with each other or with other chemotherapeutic agents or targeted therapies showed promising anti-tumor and disease-modifying effects due to their synergistic or additive mechanisms. Moreover, combination therapy may be able to reduce or delay emergence of resistance by concurrent targeting of molecular pathways essential for cellular viability or by inhibiting compensatory escape routes.

Since epigenetic therapies have repeatedly demonstrated intrinsic immune-modulatory properties in preclinical studies, combining epigenetic therapy with immunotherapy in general and immune checkpoint inhibition in particular is a promising approach in PDAC management, and is being validated in several phase I/II trials [117,118,119,145,167,199].

Like most anti-cancer agents, epigenetic therapies may not always solely target the gene, biological process or cell of interest. Such off-target effects may particularly cause excess of side effects. However, decrease of dose can reduce frequency and severity of side effects, and, as shown for DNMTi in the past, can also increase the epigenetic/reprogramming potential of epigenetic agents (while reducing its cytotoxic impact) [208]. In addition, effects that are currently considered to be off-target may eventually be desirable. For example, beside their ability to re-activate tumor suppressor genes, DNMTi can also activate the expression of other genes silenced in normal cells and encoding for endogenous retroviruses (ERVs), latent cancer testis antigens (CTAs), Alu elements and long interspersed elements (LINEs) all of which can modulate tumor cell visibility to the host immune system [209]. Recently, the hydroxamate class of HDACi showed an off-target inhibition of MBLAC2 leading to accumulation of extracellular vesicles, thus unravelling a new HDAC-independent therapeutic mechanism [210].

Nevertheless, the large-scale changes in gene expressions induced by epigenetic therapy can pose risk to normal cells, which also rely on epigenetic plasticity in their differentiation and development. In line with that, epigenetic inhibitors frequently cause hematopoietic side effects, such as thrombocytopenia, neutropenia and anemia or non-hematologic toxicities including fatigue, diarrhea, nausea and vomiting which can reach grade 3/4 severity [211,212,213]. It remains to be established to what extent more selective agents such as the DNMT1i GSK3685032 [214], the HDAC9i nanatinostat [215] or the HDAC6i ricolinostat [216] exhibit decreased toxicity by comparable or improved efficacy.

In summary, increased understanding of the role of epigenetic alterations in PDAC progression and metastasis has paved the way for several studies to discover epigenetic biomarker panels, prediction algorithms and therapeutic strategies aiming to improve the outcomes of PDAC patients. This demonstrates that we are on the verge of implementing epigenetics in the clinical management of our patients. Relevant next steps will be to establish epigenetic biomarkers for treatment stratification and monitoring in prospective studies and to identify the most promising treatment combinations for further phase III development (under special consideration of those combinations implementing immunotherapies and/or having an optimal therapeutic index). The further investigation of epigenetic biomarkers and treatments has to be performed jointly in order to allow the identification of those patients, who may most likely benefit from the respective treatment. The optimal utilization of epigenetics in diagnostics and treatment holds the promise to significantly improve the dismal prognosis of patients with PDAC.

## Figures and Tables

**Table 1:** Studies assessing the diagnostic utility of potential epigenetic liquid biopsy markers in PDAC.

Test	Type	Sample	Arms	Results	Reference
Methylation of a 5-gene panel	Diagnostic	Blood	PDAC Healthy controls	Differentiated PDAC from controls; sensitivity 76%, specificity 59%	[81]
Methylation of a 6-gene panel	Diagnostic	Blood	PDAC Chronic pancreatitis Healthy controls	Differentiated PDAC from healthy controls but not chronic pancreatitis	[82]
Hypermethylation of *NPTX2*	Diagnostic	Blood	PDAC Chronic pancreatitis Biliary stone diseases	Differentiated PDAC from chronic pancreatitis; sensitivity 80%, specificity 76%	[83]
Hypermethylation *of NPTX2* and *SPARC*	Diagnostic Prognostic	Blood	PDAC Chronic pancreatitis	Differentiated PDAC from chronic pancreatitis Associated with poor survival	[84]
Methylation of *BNC1* and *ADAMTS1*	Diagnostic	Blood	PDAC No PDAC	Differentiated PDAC from controls without PDAC; sensitivity 97.4%, specificity 91.6%	[85]
*Methylation of BNC1* and *ADAMTS1*	Diagnostic	Blood	PDAC Healthy controls	Differentiated PDAC from controls; sensitivity 81%, specificity 85%	[86]
Methylation of an 8-gene panel	Diagnostic	Blood	PDAC Chronic/acute pancreatitis No pancreatic disease	Differentiated PDAC from controls; sensitivity 76%, specificity 83%	[87]
Tissue-specific DNA methylation markers	Diagnostic	Blood	PDAC Chronic pancreatitis Healthy controls	Differentiated PDAC and pancreatitis from controls	[88]
Panel of differentially methylated regions (DMR)	Diagnostic	Blood	PDAC Other gastrointestinal cancers	Differentiated PDAC from other cancers	[89]
Methylation of a 13- gene panel + CA19-9 level	Diagnostic	Blood	PDAC Healthy controls	Detected PDAC across all stages compared to controls; at pre-set specificity 97.5%: sensitivity 92%, specificity 92%	[90]
Methylation of a 10- gene panel	Diagnostic Monitoring	Blood	PDAC Benign pancreatic cysts	Distinguished between metastatic PDAC and benign cysts; sensitivity 100%, Specificity 95% Decrease in methylation levels upon treatment	[91]
Methylation of *RUNX3* + CA19-9 level	Diagnostic	Blood	PDAC Benign pancreatic disease Healthy controls	Detected PDAC compared to other arms; sensitivity 85.5%, specificity 93.5%	[92]
Methylation of *C13orf18*, *FER1L4* and *BMP3*	Diagnostic	Pancreatic juice	PDAC IPMN with high grade dysplasia Benign disease Healthy controls	Distinguished between any stage of PDAC and controls; at pre-set specificity 86%: sensitivity 83% Identified patients with stage I or II PDAC or IPMN; at pre-set specificity 86%: sensitivity 80%	[94]
Methylation of *TBX15*, *BMP3*	Diagnostic	Pancreatic cyst fluid	PDAC High grade dysplasia Low or no dysplasia	Distinguished between PDAC and high grade dysplasia from other conditions; sensitivity and specificity above 90%	[95]
Methylation of *BMP3* and mutant *KRAS*	Diagnostic	Stool	PDAC Healthy controls	Distinguished between PDAC and controls; at pre-set specificity 90%: sensitivity 67%	[96]
Number and specific set of hypermethylated genes	Prognostic	Blood	PDAC	Differentiated between metastatic disease and earlier stages	[97]
Number of hypermethylated genes	Prognostic	Blood	PDAC	Patients with more than 10 hypermethylated genes (of 28 analyzed) had worse survival outcomes	[98]
Methylation of a predefined gene panel	Prognostic	Blood	PDAC	Overall and disease stage-specific risk models based on the methylation status of the genes analyzed	[98]
Methylation of *HOXD8* and *POU4F1*	Prognostic	Blood	PDAC	Independent prognostic marker for PFS and OS	[100]
A panel of nucleosomal biomarkers with or without CA19-9	Diagnostic	Blood	PDAC Benign pancreatic disease Healthy controls	Two models distinguished PDAC from other arms at pre-set specificity (90%); model 1 (5 nucleosomal biomarkers): sensitivity 72%; model 2 (4 nucleosomal biomarkers + CA19-9): sensitivity 92%	[103]

IPMN, intraductal papillary mucinous neoplasm; OS, overall survival; PDAC, pancreatic ductal adenocarcinoma; PFS, progression free survival.

## References

[1] Siegel R.L., Miller K.D., Fuchs H.E., Jemal A. (2022). Cancer Statistics, 2022. CA Cancer J. Clin..

[2] Schouten T.J., Daamen L.A., Dorland G., van Roessel S.R., Groot V.P., Besselink M.G., Bonsing B.A., Bosscha K., Brosens L.A.A., Busch O.R. (2022). Nationwide Validation of the 8th American Joint Committee on Cancer TNM Staging System and Five Proposed Modifications for Resected Pancreatic Cancer. Ann. Surg. Oncol..

[3] Catalano M., Aprile G., Conca R., Petrioli R., Ramello M., Roviello G. (2022). The Impact of Age, Performance Status and Comorbidities on Nab-Paclitaxel plus Gemcitabine Effectiveness in Patients with Metastatic Pancreatic Cancer. Sci. Rep..

[4] Balaban E.P., Mangu P.B., Yee N.S. (2017). Locally Advanced Unresectable Pancreatic Cancer: American Society of Clinical Oncology Clinical Practice Guideline Summary. J. Oncol. Pract..

[5] Singh R.R., O’Reilly E.M. (2020). New Treatment Strategies for Metastatic Pancreatic Ductal Adenocarcinoma. Drugs.

[6] Conroy T., Hammel P., Hebbar M., Ben Abdelghani M., Wei A.C., Raoul J.-L., Choné L., Francois E., Artru P., Biagi J.J. (2018). FOLFIRINOX or Gemcitabine as Adjuvant Therapy for Pancreatic Cancer. N. Engl. J. Med..

[7] Moskaluk C.A., Hruban R.H., Kern S.E. (1997). P16 and K-Ras Gene Mutations in the Intraductal Precursors of Human Pancreatic Adenocarcinoma. Cancer Res..

[8] DiGiuseppe J.A., Redston M.S., Yeo C.J., Kern S.E., Hruban R.H. (1995). P53-Independent Expression of the Cyclin-Dependent Kinase Inhibitor P21 in Pancreatic Carcinoma. Am. J. Pathol..

[9] Dardare J., Witz A., Merlin J.-L., Gilson P., Harlé A. (2020). SMAD4 and the TGFβ Pathway in Patients with Pancreatic Ductal Adenocarcinoma. Int. J. Mol. Sci..

[10] Bartsch D.K., Sina-Frey M., Lang S., Wild A., Gerdes B., Barth P., Kress R., Grützmann R., Colombo-Benkmann M., Ziegler A. (2002). CDKN2A Germline Mutations in Familial Pancreatic Cancer. Ann. Surg..

[11] Lomberk G., Blum Y., Nicolle R., Nair A., Gaonkar K.S., Marisa L., Mathison A., Sun Z., Yan H., Elarouci N. (2018). Distinct Epigenetic Landscapes Underlie the Pathobiology of Pancreatic Cancer Subtypes. Nat. Commun..

[12] Thompson M.J., Rubbi L., Dawson D.W., Donahue T.R., Pellegrini M. (2015). Pancreatic Cancer Patient Survival Correlates with DNA Methylation of Pancreas Development Genes. PLoS ONE.

[13] Makohon-Moore A.P., Zhang M., Reiter J.G., Bozic I., Allen B., Kundu D., Chatterjee K., Wong F., Jiao Y., Kohutek Z.A. (2017). Limited Heterogeneity of Known Driver Gene Mutations among the Metastases of Individual Patients with Pancreatic Cancer. Nat. Genet..

[14] Embuscado E.E., Laheru D., Ricci F., Yun K.J., de Boom Witzel S., Seigel A., Flickinger K., Hidalgo M., Bova G.S., Iacobuzio-Donahue C.A. (2005). Immortalizing the Complexity of Cancer Metastasis Genetic Features of Lethal Metastatic Pancreatic Cancer Obtained from Rapid Autopsy. Cancer Biol. Ther..

[15] Waddington C.H. (2012). The Epigenotype. 1942. Int. J. Epidemiol..

[16] Licht J.D., Bennett R.L. (2021). Leveraging Epigenetics to Enhance the Efficacy of Immunotherapy. Clin. Epigenetics.

[17] Wang S.S., Xu J., Ji K.Y., Hwang C.-I. (2021). Epigenetic Alterations in Pancreatic Cancer Metastasis. Biomolecules.

[18] Hayashi A., Fan J., Chen R., Ho Y., Makohon-Moore A.P., Lecomte N., Zhong Y., Hong J., Huang J., Sakamoto H. (2020). A Unifying Paradigm for Transcriptional Heterogeneity and Squamous Features in Pancreatic Ductal Adenocarcinoma. Nat. Cancer.

[19] McDonald O.G., Li X., Saunders T., Tryggvadottir R., Mentch S.J., Warmoes M.O., Word A.E., Carrer A., Salz T.H., Natsume S. (2017). Large-Scale Epigenomic Reprogramming during Pancreatic Cancer Progression Links Anabolic Glucose Metabolism to Distant Metastasis. Nat. Genet..

[20] Jones P.A., Baylin S.B. (2002). The Fundamental Role of Epigenetic Events in Cancer. Nat. Rev. Genet..

[21] Lander E.S., Linton L.M., Birren B., Nusbaum C., Zody M.C., Baldwin J., Devon K., Dewar K., Doyle M., FitzHugh W. (2001). Initial Sequencing and Analysis of the Human Genome. Nature.

[22] Jeziorska D.M., Murray R.J.S., De Gobbi M., Gaentzsch R., Garrick D., Ayyub H., Chen T., Li E., Telenius J., Lynch M. (2017). DNA Methylation of Intragenic CpG Islands Depends on Their Transcriptional Activity during Differentiation and Disease. Proc. Natl. Acad. Sci. USA.

[23] Herman J.G., Baylin S.B. (2003). Gene Silencing in Cancer in Association with Promoter Hypermethylation. N. Engl. J. Med..

[24] Wade P.A., Gegonne A., Jones P.L., Ballestar E., Aubry F., Wolffe A.P. (1999). Mi-2 Complex Couples DNA Methylation to Chromatin Remodelling and Histone Deacetylation. Nat. Genet..

[25] Okano M., Bell D.W., Haber D.A., Li E. (1999). DNA Methyltransferases Dnmt3a and Dnmt3b Are Essential for de Novo Methylation and Mammalian Development. Cell.

[26] Scourzic L., Mouly E., Bernard O.A. (2015). TET Proteins and the Control of Cytosine Demethylation in Cancer. Genome Med..

[27] Tan A.C., Jimeno A., Lin S.H., Wheelhouse J., Chan F., Solomon A., Rajeshkumar N.V., Rubio-Viqueira B., Hidalgo M. (2009). Characterizing DNA Methylation Patterns in Pancreatic Cancer Genome. Mol. Oncol..

[28] Cancer Genome Atlas Research Network (2017). Integrated Genomic Characterization of Pancreatic Ductal Adenocarcinoma. Cancer Cell.

[29] Zhu J., Yang Y., Kisiel J.B., Mahoney D.W., Michaud D.S., Guo X., Taylor W.R., Shu X.-O., Shu X., Liu D. (2021). Integrating Genome and Methylome Data to Identify Candidate DNA Methylation Biomarkers for Pancreatic Cancer Risk. Cancer Epidemiol. Biomarkers Prev..

[30] Ozturk H., Cingoz H., Tufan T., Yang J., Adair S.J., Tummala K.S., Kuscu C., Kinali M., Comertpay G., Nagdas S. (2022). ISL2 Is a Putative Tumor Suppressor Whose Epigenetic Silencing Reprograms the Metabolism of Pancreatic Cancer. Dev. Cell.

[31] Sato N., Maitra A., Fukushima N., van Heek N.T., Matsubayashi H., Iacobuzio-Donahue C.A., Rosty C., Goggins M. (2003). Frequent Hypomethylation of Multiple Genes Overexpressed in Pancreatic Ductal Adenocarcinoma. Cancer Res..

[32] Espinet E., Gu Z., Imbusch C.D., Giese N.A., Büscher M., Safavi M., Weisenburger S., Klein C., Vogel V., Falcone M. (2021). Aggressive PDACs Show Hypomethylation of Repetitive Elements and the Execution of an Intrinsic IFN Program Linked to a Ductal Cell of Origin. Cancer Discov..

[33] Eyres M., Lanfredini S., Xu H., Burns A., Blake A., Willenbrock F., Goldin R., Hughes D., Hughes S., Thapa A. (2021). TET2 Drives 5hmc Marking of GATA6 and Epigenetically Defines Pancreatic Ductal Adenocarcinoma Transcriptional Subtypes. Gastroenterology.

[34] Cheng C., Huang C., Ma T.-T., Bian E.-B., He Y., Zhang L., Li J. (2014). SOCS1 Hypermethylation Mediated by DNMT1 Is Associated with Lipopolysaccharide-Induced Inflammatory Cytokines in Macrophages. Toxicol. Lett..

[35] Tang R.-Z., Zhu J.-J., Yang F.-F., Zhang Y.-P., Xie S.-A., Liu Y.-F., Yao W.-J., Pang W., Han L.-L., Kong W. (2019). DNA Methyltransferase 1 and Krüppel-like Factor 4 Axis Regulates Macrophage Inflammation and Atherosclerosis. J. Mol. Cell Cardiol..

[36] Zhang M., Pan X., Fujiwara K., Jurcak N., Muth S., Zhou J., Xiao Q., Li A., Che X., Li Z. (2021). Pancreatic Cancer Cells Render Tumor-Associated Macrophages Metabolically Reprogrammed by a GARP and DNA Methylation-Mediated Mechanism. Signal Transduct. Target Ther..

[37] Xiao Q., Zhou D., Rucki A.A., Williams J., Zhou J., Mo G., Murphy A., Fujiwara K., Kleponis J., Salman B. (2016). Cancer-Associated Fibroblasts in Pancreatic Cancer Are Reprogrammed by Tumor-Induced Alterations in Genomic DNA Methylation. Cancer Res..

[38] Yu M., Hazelton W.D., Luebeck G.E., Grady W.M. (2020). Epigenetic Aging: More Than Just a Clock When It Comes to Cancer. Cancer Res..

[39] Bell C.G., Lowe R., Adams P.D., Baccarelli A.A., Beck S., Bell J.T., Christensen B.C., Gladyshev V.N., Heijmans B.T., Horvath S. (2019). DNA Methylation Aging Clocks: Challenges and Recommendations. Genome Biol..

[40] Rawla P., Sunkara T., Gaduputi V. (2019). Epidemiology of Pancreatic Cancer: Global Trends, Etiology and Risk Factors. World J. Oncol..

[41] Cancer of the Pancreas—Cancer Stat Facts. https://seer.cancer.gov/statfacts/html/pancreas.html.

[42] Raffenne J., Martin F.A., Nicolle R., Konta M., Blum Y., Torrisani J., Puleo F., Bachet J.B., Svrcek M., Bardier-Dupas A. (2021). Pancreatic Ductal Adenocarcinoma Arising in Young and Old Patients Displays Similar Molecular Features. Cancers.

[43] Chung M., Ruan M., Zhao N., Koestler D.C., De Vivo I., Kelsey K.T., Michaud D.S. (2021). DNA Methylation Ageing Clocks and Pancreatic Cancer Risk: Pooled Analysis of Three Prospective Nested Case-Control Studies. Epigenetics.

[44] Zhao Z., Shilatifard A. (2019). Epigenetic Modifications of Histones in Cancer. Genome Biol..

[45] Köenig A., Linhart T., Schlengemann K., Reutlinger K., Wegele J., Adler G., Singh G., Hofmann L., Kunsch S., Büch T. (2010). NFAT-Induced Histone Acetylation Relay Switch Promotes c-Myc-Dependent Growth in Pancreatic Cancer Cells. Gastroenterology.

[46] Mees S.T., Mardin W.A., Wendel C., Baeumer N., Willscher E., Senninger N., Schleicher C., Colombo-Benkmann M., Haier J. (2010). EP300--a MiRNA-Regulated Metastasis Suppressor Gene in Ductal Adenocarcinomas of the Pancreas. Int. J. Cancer.

[47] Cai M.-H., Xu X.-G., Yan S.-L., Sun Z., Ying Y., Wang B.-K., Tu Y.-X. (2018). Depletion of HDAC1, 7 and 8 by Histone Deacetylase Inhibition Confers Elimination of Pancreatic Cancer Stem Cells in Combination with Gemcitabine. Sci. Rep..

[48] Klieser E., Swierczynski S., Mayr C., Schmidt J., Neureiter D., Kiesslich T., Illig R. (2015). Role of Histone Deacetylases in Pancreas: Implications for Pathogenesis and Therapy. World J. Gastrointest. Oncol..

[49] Brand M., Measures A.R., Measures A.M., Wilson B.G., Cortopassi W.A., Alexander R., Höss M., Hewings D.S., Rooney T.P.C., Paton R.S. (2015). Small Molecule Inhibitors of Bromodomain-Acetyl-Lysine Interactions. ACS Chem. Biol..

[50] Donati B., Lorenzini E., Ciarrocchi A. (2018). BRD4 and Cancer: Going beyond Transcriptional Regulation. Mol. Cancer.

[51] Yan J., Diaz J., Jiao J., Wang R., You J. (2011). Perturbation of BRD4 Protein Function by BRD4-NUT Protein Abrogates Cellular Differentiation in NUT Midline Carcinoma. J. Biol. Chem..

[52] Junwei S., Vakoc C.R. (2014). The Mechanisms behind the Therapeutic Activity of BET Bromodomain Inhibition. Mol. Cell.

[53] Greer E.L., Shi Y. (2012). Histone Methylation: A Dynamic Mark in Health, Disease and Inheritance. Nat. Rev. Genet..

[54] Chen Y., Ren B., Yang J., Wang H., Yang G., Xu R., You L., Zhao Y. (2020). The Role of Histone Methylation in the Development of Digestive Cancers: A Potential Direction for Cancer Management. Signal Transduct. Target Ther..

[55] Benitz S., Straub T., Mahajan U.M., Mutter J., Czemmel S., Unruh T., Wingerath B., Deubler S., Fahr L., Cheng T. (2019). Ring1b-Dependent Epigenetic Remodelling Is an Essential Prerequisite for Pancreatic Carcinogenesis. Gut.

[56] Andricovich J., Perkail S., Kai Y., Casasanta N., Peng W., Tzatsos A. (2018). Loss of KDM6A Activates Super-Enhancers to Induce Gender-Specific Squamous-like Pancreatic Cancer and Confers Sensitivity to BET Inhibitors. Cancer Cell.

[57] Rao R.A., Dhele N., Cheemadan S., Ketkar A., Jayandharan G.R., Palakodeti D., Rampalli S. (2015). Ezh2 Mediated H3K27me3 Activity Facilitates Somatic Transition during Human Pluripotent Reprogramming. Sci. Rep..

[58] Ougolkov A.V., Bilim V.N., Billadeau D.D. (2008). Regulation of Pancreatic Tumor Cell Proliferation and Chemoresistance by the Histone Methyltransferase EZH2. Clin. Cancer Res..

[59] Patil S., Steuber B., Kopp W., Kari V., Urbach L., Wang X., Küffer S., Bohnenberger H., Spyropoulou D., Zhang Z. (2020). EZH2 Regulates Pancreatic Cancer Subtype Identity and Tumor Progression via Transcriptional Repression of GATA6. Cancer Res..

[60] Adamska A., Domenichini A., Falasca M. (2017). Pancreatic Ductal Adenocarcinoma: Current and Evolving Therapies. Int. J. Mol. Sci..

[61] Yachida S., White C.M., Naito Y., Zhong Y., Brosnan J.A., Macgregor-Das A.M., Morgan R.A., Saunders T., Laheru D.A., Herman J.M. (2012). Clinical Significance of the Genetic Landscape of Pancreatic Cancer and Implications for Identification of Potential Long Term Survivors. Clin. Cancer Res..

[62] Miquel M., Zhang S., Pilarsky C. (2021). Pre-Clinical Models of Metastasis in Pancreatic Cancer. Front. Cell Dev. Biol..

[63] Toll A.D., Dasgupta A., Potoczek M., Yeo C.J., Kleer C.G., Brody J.R., Witkiewicz A.K. (2010). Implications of Enhancer of Zeste Homologue 2 Expression in Pancreatic Ductal Adenocarcinoma. Hum. Pathol..

[64] Han T., Jiao F., Hu H., Yuan C., Wang L., Jin Z.-L., Song W., Wang L.-W. (2016). EZH2 Promotes Cell Migration and Invasion but Not Alters Cell Proliferation by Suppressing E-Cadherin, Partly through Association with MALAT-1 in Pancreatic Cancer. Oncotarget.

[65] von Burstin J., Eser S., Paul M.C., Seidler B., Brandl M., Messer M., von Werder A., Schmidt A., Mages J., Pagel P. (2009). E-Cadherin Regulates Metastasis of Pancreatic Cancer in Vivo and Is Suppressed by a SNAIL/HDAC1/HDAC2 Repressor Complex. Gastroenterology.

[66] Aghdassi A., Sendler M., Guenther A., Mayerle J., Behn C.-O., Heidecke C.-D., Friess H., Büchler M., Evert M., Lerch M.M. (2012). Recruitment of Histone Deacetylases HDAC1 and HDAC2 by the Transcriptional Repressor ZEB1 Downregulates E-Cadherin Expression in Pancreatic Cancer. Gut.

[67] Song Y., Washington M.K., Crawford H.C. (2010). Loss of FOXA1/2 Is Essential for the Epithelial-to-Mesenchymal Transition in Pancreatic Cancer. Cancer Res..

[68] Roe J.-S., Hwang C.-I., Somerville T.D.D., Milazzo J.P., Lee E.J., Da Silva B., Maiorino L., Tiriac H., Young C.M., Miyabayashi K. (2017). Enhancer Reprogramming Promotes Pancreatic Cancer Metastasis. Cell.

[69] Sato N., Parker A.R., Fukushima N., Miyagi Y., Iacobuzio-Donahue C.A., Eshleman J.R., Goggins M. (2005). Epigenetic Inactivation of TFPI-2 as a Common Mechanism Associated with Growth and Invasion of Pancreatic Ductal Adenocarcinoma. Oncogene.

[70] Sato N., Fukushima N., Chang R., Matsubayashi H., Goggins M. (2006). Differential and Epigenetic Gene Expression Profiling Identifies Frequent Disruption of the RELN Pathway in Pancreatic Cancers. Gastroenterology.

[71] Nones K., Waddell N., Song S., Patch A.-M., Miller D., Johns A., Wu J., Kassahn K.S., Wood D., Bailey P. (2014). Genome-Wide DNA Methylation Patterns in Pancreatic Ductal Adenocarcinoma Reveal Epigenetic Deregulation of SLIT-ROBO, ITGA2 and MET Signaling. Int. J. Cancer.

[72] Zhang Z.-M., Wang J.-S., Zulfiqar H., Lv H., Dao F.-Y., Lin H. (2020). Early Diagnosis of Pancreatic Ductal Adenocarcinoma by Combining Relative Expression Orderings with Machine-Learning Method. Front. Cell Dev. Biol..

[73] Orth M., Metzger P., Gerum S., Mayerle J., Schneider G., Belka C., Schnurr M., Lauber K. (2019). Pancreatic Ductal Adenocarcinoma: Biological Hallmarks, Current Status, and Future Perspectives of Combined Modality Treatment Approaches. Radiat. Oncol..

[74] Zhang L., Sanagapalli S., Stoita A. (2018). Challenges in Diagnosis of Pancreatic Cancer. World J. Gastroenterol..

[75] Wan J.C.M., Massie C., Garcia-Corbacho J., Mouliere F., Brenton J.D., Caldas C., Pacey S., Baird R., Rosenfeld N. (2017). Liquid Biopsies Come of Age: Towards Implementation of Circulating Tumour DNA. Nat. Rev. Cancer.

[76] George B., Haberberger J., Ferguson N.L., Gjoerup O., McGregor K., Hendifar A.E., Laheru D.A., Weekes C.D., Ross J.S., Hemmerich A. (2021). Correlation between Comprehensive Genomic Profiling (CGP) Utilizing Tissue-Based Testing (T-CGP) and Cell-Free DNA (CfDNA) in Patients (Pts) with Pancreatic Ductal Adenocarcinoma (PDAC). J. Clin. Oncol..

[77] Hipp J., Hussung S., Timme-Bronsert S., Boerries M., Biesel E., Fichtner-Feigl S., Fritsch R., Wittel U.A. (2021). Perioperative Cell-Free Mutant KRAS Dynamics in Patients with Pancreatic Cancer. Br. J. Surg..

[78] Hussung S., Akhoundova D., Hipp J., Follo M., Klar R.F.U., Philipp U., Scherer F., von Bubnoff N., Duyster J., Boerries M. (2021). Longitudinal Analysis of Cell-Free Mutated KRAS and CA 19-9 Predicts Survival Following Curative Resection of Pancreatic Cancer. BMC Cancer.

[79] Wehrle J., Philipp U., Jolic M., Follo M., Hussung S., Waldeck S., Deuter M., Rassner M., Braune J., Rawluk J. (2020). Personalized Treatment Selection and Disease Monitoring Using Circulating Tumor DNA Profiling in Real-World Cancer Patient Management. Diagnostics.

[80] Kulemann B., Rösch S., Seifert S., Timme S., Bronsert P., Seifert G., Martini V., Kuvendjiska J., Glatz T., Hussung S. (2017). Pancreatic Cancer: Circulating Tumor Cells and Primary Tumors Show Heterogeneous KRAS Mutations. Sci. Rep..

[81] Melnikov A.A., Scholtens D., Talamonti M.S., Bentrem D.J., Levenson V.V. (2009). Methylation Profile of Circulating Plasma DNA in Patients with Pancreatic Cancer. J. Surg. Oncol..

[82] Park J.W., Baek I.H., Kim Y.T. (2012). Preliminary Study Analyzing the Methylated Genes in the Plasma of Patients with Pancreatic Cancer. Scand. J. Surg..

[83] Park J.K., Ryu J.K., Yoon W.J., Lee S.H., Lee G.Y., Jeong K.-S., Kim Y.-T., Yoon Y.B. (2012). The Role of Quantitative NPTX2 Hypermethylation as a Novel Serum Diagnostic Marker in Pancreatic Cancer. Pancreas.

[84] Singh N., Rashid S., Rashid S., Dash N.R., Gupta S., Saraya A. (2020). Clinical Significance of Promoter Methylation Status of Tumor Suppressor Genes in Circulating DNA of Pancreatic Cancer Patients. J. Cancer Res. Clin. Oncol..

[85] Eissa M.A.L., Lerner L., Abdelfatah E., Shankar N., Canner J.K., Hasan N.M., Yaghoobi V., Huang B., Kerner Z., Takaesu F. (2019). Promoter Methylation of ADAMTS1 and BNC1 as Potential Biomarkers for Early Detection of Pancreatic Cancer in Blood. Clin. Epigenetics.

[86] Yi J.M., Guzzetta A.A., Bailey V.J., Downing S.R., Van Neste L., Chiappinelli K.B., Keeley B.P., Stark A., Herrera A., Wolfgang C. (2013). Novel Methylation Biomarker Panel for the Early Detection of Pancreatic Cancer. Clin. Cancer Res..

[87] Henriksen S.D., Madsen P.H., Larsen A.C., Johansen M.B., Drewes A.M., Pedersen I.S., Krarup H., Thorlacius-Ussing O. (2016). Cell-Free DNA Promoter Hypermethylation in Plasma as a Diagnostic Marker for Pancreatic Adenocarcinoma. Clin. Epigenetics.

[88] Lehmann-Werman R., Neiman D., Zemmour H., Moss J., Magenheim J., Vaknin-Dembinsky A., Rubertsson S., Nellgård B., Blennow K., Zetterberg H. (2016). Identification of Tissue-Specific Cell Death Using Methylation Patterns of Circulating DNA. Proc. Natl. Acad. Sci. USA.

[89] Kandimalla R., Xu J., Link A., Matsuyama T., Yamamura K., Parker M.I., Uetake H., Balaguer F., Borazanci E., Tsai S. (2021). EpiPanGI Dx: A Cell-Free DNA Methylation Fingerprint for the Early Detection of Gastrointestinal Cancers. Clin. Cancer Res..

[90] Majumder S., Taylor W.R., Foote P.H., Berger C.K., Wu C.W., Mahoney D.W., Bamlet W.R., Burger K.N., Postier N., de la Fuente J. (2021). High Detection Rates of Pancreatic Cancer Across Stages by Plasma Assay of Novel Methylated DNA Markers and CA19-9. Clin. Cancer Res..

[91] Vrba L., Futscher B.W., Oshiro M., Watts G.S., Menashi E., Hu C., Hammad H., Pennington D.R., Golconda U., Gavini H. (2022). Liquid Biopsy, Using a Novel DNA Methylation Signature, Distinguishes Pancreatic Adenocarcinoma from Benign Pancreatic Disease. Clin. Epigenetics.

[92] Fujimoto Y., Suehiro Y., Kaino S., Suenaga S., Tsuyama T., Matsui H., Higaki S., Fujii I., Suzuki C., Hoshida T. (2021). Combination of CA19-9 and Blood Free-Circulating Methylated RUNX3 May Be Useful to Diagnose Stage I Pancreatic Cancer. Oncology.

[93] Henriksen S.D., Thorlacius-Ussing O. (2021). Cell-Free DNA Methylation as Blood-Based Biomarkers for Pancreatic Adenocarcinoma-A Literature Update. Epigenomes.

[94] Majumder S., Raimondo M., Taylor W.R., Yab T.C., Berger C.K., Dukek B.A., Cao X., Foote P.H., Wu C.W., Devens M.E. (2020). Methylated DNA in Pancreatic Juice Distinguishes Patients with Pancreatic Cancer from Controls. Clin. Gastroenterol. Hepatol..

[95] Majumder S., Taylor W.R., Yab T.C., Berger C.K., Dukek B.A., Cao X., Foote P.H., Wu C.W., Mahoney D.W., Aslanian H.R. (2019). Novel Methylated DNA Markers Discriminate Advanced Neoplasia in Pancreatic Cysts: Marker Discovery, Tissue Validation, and Cyst Fluid Testing. Am. J. Gastroenterol..

[96] Kisiel J.B., Yab T.C., Taylor W.R., Chari S.T., Petersen G.M., Mahoney D.W., Ahlquist D.A. (2012). Stool DNA Testing for the Detection of Pancreatic Cancer: Assessment of Methylation Marker Candidates. Cancer.

[97] Henriksen S.D., Madsen P.H., Larsen A.C., Johansen M.B., Pedersen I.S., Krarup H., Thorlacius-Ussing O. (2017). Promoter Hypermethylation in Plasma-Derived Cell-Free DNA as a Prognostic Marker for Pancreatic Adenocarcinoma Staging. Int. J. Cancer.

[98] Henriksen S.D., Madsen P.H., Larsen A.C., Johansen M.B., Pedersen I.S., Krarup H., Thorlacius-Ussing O. (2017). Cell-Free DNA Promoter Hypermethylation in Plasma as a Predictive Marker for Survival of Patients with Pancreatic Adenocarcinoma. Oncotarget.

[99] Zhang Z., Zhu R., Sun W., Wang J., Liu J. (2021). Analysis of Methylation-driven Genes in Pancreatic Ductal Adenocarcinoma for Predicting Prognosis. J. Cancer.

[100] Pietrasz D., Wang-Renault S., Taieb J., Dahan L., Postel M., Durand-Labrunie J., Le Malicot K., Mulot C., Rinaldi Y., Phelip J.-M. (2022). Prognostic Value of Circulating Tumour DNA in Metastatic Pancreatic Cancer Patients: Post-Hoc Analyses of Two Clinical Trials. Br. J. Cancer.

[101] Corcoran R.B., Chabner B.A. (2018). Application of Cell-Free DNA Analysis to Cancer Treatment. N. Engl. J. Med..

[102] Holdenrieder S., Stieber P. (2009). Clinical Use of Circulating Nucleosomes. Crit. Rev. Clin. Lab. Sci..

[103] Bauden M., Pamart D., Ansari D., Herzog M., Eccleston M., Micallef J., Andersson B., Andersson R. (2015). Circulating Nucleosomes as Epigenetic Biomarkers in Pancreatic Cancer. Clin. Epigenetics.

[104] Mottini C., Tomihara H., Carrella D., Lamolinara A., Iezzi M., Huang J.K., Amoreo C.A., Buglioni S., Manni I., Robinson F.S. (2019). Predictive Signatures Inform the Effective Repurposing of Decitabine to Treat KRAS-Dependent Pancreatic Ductal Adenocarcinoma. Cancer Res..

[105] A Proof-of-Concept, Biomarker-Driven, Phase-II Clinical Trial to Explore the Activity of Decitabine Repurposing Against Advanced, Refractory, KRAS-Dependent Pancreatic Ductal Adenocarcinoma (PDAC): The ORIENTATE Trial. https://clinicaltrials.gov/ct2/show/NCT05360264.

[106] Tsuda M., Fukuda A., Kawai M., Araki O., Seno H. (2021). The Role of the SWI/SNF Chromatin Remodeling Complex in Pancreatic Ductal Adenocarcinoma. Cancer Sci..

[107] Italiano A., Soria J.-C., Toulmonde M., Michot J.-M., Lucchesi C., Varga A., Coindre J.-M., Blakemore S.J., Clawson A., Suttle B. (2018). Tazemetostat, an EZH2 Inhibitor, in Relapsed or Refractory B-Cell Non-Hodgkin Lymphoma and Advanced Solid Tumours: A First-in-Human, Open-Label, Phase 1 Study. Lancet Oncol..

[108] Chan-Penebre E., Armstrong K., Drew A., Grassian A.R., Feldman I., Knutson S.K., Kuplast-Barr K., Roche M., Campbell J., Ho P. (2017). Selective Killing of SMARCA2- and SMARCA4-Deficient Small Cell Carcinoma of the Ovary, Hypercalcemic Type Cells by Inhibition of EZH2: In Vitro and In Vivo Preclinical Models. Mol. Cancer Ther..

[109] Stomper J., Rotondo J.C., Greve G., Lübbert M. (2021). Hypomethylating Agents (HMA) for the Treatment of Acute Myeloid Leukemia and Myelodysplastic Syndromes: Mechanisms of Resistance and Novel HMA-Based Therapies. Leukemia.

[110] DiNardo C.D., Pratz K., Pullarkat V., Jonas B.A., Arellano M., Becker P.S., Frankfurt O., Konopleva M., Wei A.H., Kantarjian H.M. (2019). Venetoclax Combined with Decitabine or Azacitidine in Treatment-Naive, Elderly Patients with Acute Myeloid Leukemia. Blood.

[111] Becker H., Pfeifer D., Ihorst G., Pantic M., Wehrle J., Rüter B.H., Bullinger L., Hackanson B., Germing U., Kuendgen A. (2020). Monosomal Karyotype and Chromosome 17p Loss or TP53 Mutations in Decitabine-Treated Patients with Acute Myeloid Leukemia. Ann. Hematol..

[112] Gailhouste L., Liew L.C., Hatada I., Nakagama H., Ochiya T. (2018). Epigenetic Reprogramming Using 5-Azacytidine Promotes an Anti-Cancer Response in Pancreatic Adenocarcinoma Cells. Cell Death Dis..

[113] A Phase 1b Study: Treatment of Refractory Pancreatic Adenocarcinoma and Advanced Soft Tissue or Bone Sarcomas Using Decitabine Combined with Gemcitabine. https://clinicaltrials.gov/ct2/show/NCT02959164.

[114] Shakya R., Gonda T., Quante M., Salas M., Kim S., Brooks J., Hirsch S., Davies J., Cullo A., Olive K. (2013). Hypomethylating Therapy in an Aggressive Stroma-Rich Model of Pancreatic Carcinoma. Cancer Res..

[115] Gonda T.A., Fang J., Salas M., Do C., Hsu E., Zhukovskaya A., Siegel A., Takahashi R., Lopez-Bujanda Z.A., Drake C.G. (2020). A DNA Hypomethylating Drug Alters the Tumor Microenvironment and Improves the Effectiveness of Immune Checkpoint Inhibitors in a Mouse Model of Pancreatic Cancer. Cancer Res..

[116] Ebelt N.D., Zuniga E., Johnson B.L., Diamond D.J., Manuel E.R. (2020). 5-Azacytidine Potentiates Anti-Tumor Immunity in a Model of Pancreatic Ductal Adenocarcinoma. Front. Immunol..

[117] Safyan R.A., Manji G.A., Lee S.M., Silva R., Bates S.E., White R.A., Jamison J.K.R., Bass A.J., Schwartz G.K., Oberstein P.E. (2022). Phase 2 Study of Azacitidine (AZA) plus Pembrolizumab (Pembro) as Second-Line Treatment in Patients with Advanced Pancreatic Ductal Adenocarcinoma. J. Clin. Oncol..

[118] A Multicenter, Phase I/II Study of Sequential Epigenetic and Immune Targeting in Combination with Nab-Paclitaxel/Gemcitabine in Patients with Advanced Pancreatic Ductal Adenocarcinoma. https://clinicaltrials.gov/ct2/show/NCT04257448.

[119] Algaze S., Hanna D.L., Azad N.S., Thomas J.S., Iqbal S., Habib D., Ning Y., Barzi A., Patel R., Lenz H.-J. (2022). A Phase Ib Study of Guadecitabine and Durvalumab in Patients with Advanced Hepatocellular Carcinoma, Pancreatic Adenocarcinoma, and Biliary Cancers. J. Clin. Oncol..

[120] Mahfouz R.Z., Jankowska A., Ebrahem Q., Gu X., Visconte V., Tabarroki A., Terse P., Covey J., Chan K., Ling Y. (2013). Increased CDA Expression/Activity in Males Contributes to Decreased Cytidine Analogue Half-Life and Likely Contributes to Worse Outcomes with 5-Azacytidine or Decitabine Therapy. Clin. Cancer Res..

[121] Sohal D., Krishnamurthi S., Tohme R., Gu X., Lindner D., Landowski T.H., Pink J., Radivoyevitch T., Fada S., Lee Z. (2020). A Pilot Clinical Trial of the Cytidine Deaminase Inhibitor Tetrahydrouridine Combined with Decitabine to Target DNMT1 in Advanced, Chemorefractory Pancreatic Cancer. Am. J. Cancer Res..

[122] P53/P16-Independent Epigenetic Therapy with Oral Decitabine/Tetrahydrouridine for Advanced Pancreatic Cancer That Has Progressed through One or More Lines of Therapy. https://clinicaltrials.gov/ct2/show/NCT02847000.

[123] Heumann T.R., Baretti M., Sugar E., Durhman J., Liden S., Miles T., Lopez-Vidal T.Y., Leatherman J., Sharma A., Ahuja N. (2021). 1470P Oral Azacitidine (CC-486) in Patients with Resected Pancreatic Adenocarcinoma at High Risk for Recurrence. Ann. Oncol..

[124] Von Hoff D.D., Rasco D.W., Heath E.I., Munster P.N., Schellens J.H.M., Isambert N., Le Tourneau C., O’Neil B., Mathijssen R.H.J., Lopez-Martin J.A. (2018). Phase I Study of CC-486 Alone and in Combination with Carboplatin or Nab-Paclitaxel in Patients with Relapsed or Refractory Solid Tumors. Clin. Cancer Res..

[125] Damaskos C., Garmpis N., Karatzas T., Nikolidakis L., Kostakis I.D., Garmpi A., Karamaroudis S., Boutsikos G., Damaskou Z., Kostakis A. (2015). Histone Deacetylase (HDAC) Inhibitors: Current Evidence for Therapeutic Activities in Pancreatic Cancer. Anticancer Res..

[126] García-Morales P., Gómez-Martínez A., Carrato A., Martínez-Lacaci I., Barberá V.M., Soto J.L., Carrasco-García E., Menéndez-Gutierrez M.P., Castro-Galache M.D., Ferragut J.A. (2005). Histone Deacetylase Inhibitors Induced Caspase-Independent Apoptosis in Human Pancreatic Adenocarcinoma Cell Lines. Mol. Cancer Ther..

[127] Tiffon C. (2018). Histone Deacetylase Inhibition Restores Expression of Hypoxia-Inducible Protein NDRG1 in Pancreatic Cancer. Pancreas.

[128] Maietta I., Martínez-Pérez A., Álvarez R., De Lera Á.R., González-Fernández Á., Simón-Vázquez R. (2022). Synergistic Antitumoral Effect of Epigenetic Inhibitors and Gemcitabine in Pancreatic Cancer Cells. Pharmaceuticals.

[129] Bai J., Demirjian A., Sui J., Marasco W., Callery M.P. (2006). Histone Deacetylase Inhibitor Trichostatin A and Proteasome Inhibitor PS-341 Synergistically Induce Apoptosis in Pancreatic Cancer Cells. Biochem. Biophys. Res. Commun..

[130] Lassen U., Molife L.R., Sorensen M., Engelholm S.-A., Vidal L., Sinha R., Penson R.T., Buhl-Jensen P., Crowley E., Tjornelund J. (2010). A Phase I Study of the Safety and Pharmacokinetics of the Histone Deacetylase Inhibitor Belinostat Administered in Combination with Carboplatin and/or Paclitaxel in Patients with Solid Tumours. Br. J. Cancer.

[131] Millward M., Price T., Townsend A., Sweeney C., Spencer A., Sukumaran S., Longenecker A., Lee L., Lay A., Sharma G. (2012). Phase 1 Clinical Trial of the Novel Proteasome Inhibitor Marizomib with the Histone Deacetylase Inhibitor Vorinostat in Patients with Melanoma, Pancreatic and Lung Cancer Based on in Vitro Assessments of the Combination. Invest. New Drugs.

[132] Jones S.F., Infante J.R., Spigel D.R., Peacock N.W., Thompson D.S., Greco F.A., McCulloch W., Burris H.A. (2012). Phase 1 Results from a Study of Romidepsin in Combination with Gemcitabine in Patients with Advanced Solid Tumors. Cancer Invest..

[133] Richards D.A., Boehm K.A., Waterhouse D.M., Wagener D.J., Krishnamurthi S.S., Rosemurgy A., Grove W., Macdonald K., Gulyas S., Clark M. (2006). Gemcitabine plus CI-994 Offers No Advantage over Gemcitabine Alone in the Treatment of Patients with Advanced Pancreatic Cancer: Results of a Phase II Randomized, Double-Blind, Placebo-Controlled, Multicenter Study. Ann. Oncol..

[134] Wang H., Cao Q., Dudek A.Z. (2012). Phase II Study of Panobinostat and Bortezomib in Patients with Pancreatic Cancer Progressing on Gemcitabine-Based Therapy. Anticancer Res..

[135] Deming D.A., Ninan J., Bailey H.H., Kolesar J.M., Eickhoff J., Reid J.M., Ames M.M., McGovern R.M., Alberti D., Marnocha R. (2014). A Phase I Study of Intermittently Dosed Vorinostat in Combination with Bortezomib in Patients with Advanced Solid Tumors. Invest. New Drugs.

[136] Iwahashi S., Utsunomiya T., Imura S., Morine Y., Ikemoto T., Arakawa Y., Saito Y., Ishikawa D., Shimada M. (2014). Effects of Valproic Acid in Combination with S-1 on Advanced Pancreatobiliary Tract Cancers: Clinical Study Phases I/II. Anticancer Res..

[137] Chan E., Chiorean E.G., O’Dwyer P.J., Gabrail N.Y., Alcindor T., Potvin D., Chao R., Hurwitz H. (2018). Phase I/II Study of Mocetinostat in Combination with Gemcitabine for Patients with Advanced Pancreatic Cancer and Other Advanced Solid Tumors. Cancer Chemother. Pharmacol..

[138] Ikeda M., Ohno I., Ueno H., Mitsunaga S., Hashimoto Y., Okusaka T., Kondo S., Sasaki M., Sakamoto Y., Takahashi H. (2019). Phase I Study of Resminostat, an HDAC Inhibitor, Combined with S-1 in Patients with Pre-Treated Biliary Tract or Pancreatic Cancer. Invest. New Drugs.

[139] Chan E., Arlinghaus L.R., Cardin D.B., Goff L., Berlin J.D., Parikh A., Abramson R.G., Yankeelov T.E., Hiebert S., Merchant N. (2016). Phase I Trial of Vorinostat Added to Chemoradiation with Capecitabine in Pancreatic Cancer. Radiother. Oncol..

[140] NPI-0052 and Vorinostat in Patients with Non-Small Cell Lung Cancer, Pancreatic Cancer, Melanoma or Lymphoma. https://clinicaltrials.gov/ct2/show/NCT00667082.

[141] A Phase I/II Study of Romidepsin (Depsipeptide) in Combination with Gemcitabine in Patients with Pancreatic and Other Advanced Solid Tumors. https://clinicaltrials.gov/ct2/show/NCT00379639.

[142] Connolly R.M., Laille E., Vaishampayan U., Chung V., Kelly K., Dowlati A., Alese O.B., Harvey R.D., Haluska P., Siu L.L. (2020). Phase I and Pharmacokinetic Study of Romidepsin in Patients with Cancer and Hepatic Dysfunction: A National Cancer Institute Organ Dysfunction Working Group Study. Clin. Cancer Res..

[143] Setiadi A.F., Omilusik K., David M.D., Seipp R.P., Hartikainen J., Gopaul R., Choi K.B., Jefferies W.A. (2008). Epigenetic Enhancement of Antigen Processing and Presentation Promotes Immune Recognition of Tumors. Cancer Res..

[144] Christmas B.J., Rafie C.I., Hopkins A.C., Scott B.A., Ma H.S., Cruz K.A., Woolman S., Armstrong T.D., Connolly R.M., Azad N.A. (2018). Entinostat Converts Immune-Resistant Breast and Pancreatic Cancers into Checkpoint-Responsive Tumors by Reprogramming Tumor-Infiltrating MDSCs. Cancer Immunol. Res..

[145] A Phase 2 Clinical Trial of Entinostat in Combination with Nivolumab for Patients with Previously Treated Unresectable or Metastatic Cholangiocarcinoma and Pancreatic Adenocarcinoma. https://clinicaltrials.gov/ct2/show/NCT03250273.

[146] Poklepovic A.S., Fields E.C., Bandyopadhyay D., Tombes M.B., Kmieciak M., McGuire W.P., Gordon S.W., Kaplan B.J., Myers J.L., Matin K. (2021). A Phase 1 Study of Neoadjuvant Chemotherapy Followed by Concurrent Chemoradiation with Gemcitabine, Sorafenib, and Vorinostat in Pancreatic Cancer. J. Clin. Oncol..

[147] Streubel G., Schrepfer S., Kallus H., Parnitzke U., Wulff T., Hermann F., Borgmann M., Hamm S. (2021). Histone Deacetylase Inhibitor Resminostat in Combination with Sorafenib Counteracts Platelet-Mediated pro-Tumoral Effects in Hepatocellular Carcinoma. Sci. Rep..

[148] Ischenko I., Petrenko O., Hayman M.J. (2015). A MEK/PI3K/HDAC Inhibitor Combination Therapy for KRAS Mutant Pancreatic Cancer Cells. Oncotarget.

[149] Wang Z., Hausmann S., Lyu R., Li T.-M., Lofgren S.M., Flores N.M., Fuentes M.E., Caporicci M., Yang Z., Meiners M.J. (2020). SETD5-Coordinated Chromatin Reprogramming Regulates Adaptive Resistance to Targeted Pancreatic Cancer Therapy. Cancer Cell.

[150] Sanz M.A., Fenaux P., Tallman M.S., Estey E.H., Löwenberg B., Naoe T., Lengfelder E., Döhner H., Burnett A.K., Chen S.-J. (2019). Management of Acute Promyelocytic Leukemia: Updated Recommendations from an Expert Panel of the European LeukemiaNet. Blood.

[151] Lübbert M., Grishina O., Schmoor C., Schlenk R.F., Jost E., Crysandt M., Heuser M., Thol F., Salih H.R., Schittenhelm M.M. (2020). Valproate and Retinoic Acid in Combination with Decitabine in Elderly Nonfit Patients with Acute Myeloid Leukemia: Results of a Multicenter, Randomized, 2 × 2, Phase II Trial. J. Clin. Oncol..

[152] Meier R., Greve G., Zimmer D., Bresser H., Berberich B., Langova R., Stomper J., Rubarth A., Feuerbach L., Lipka D.B. (2022). The Antileukemic Activity of Decitabine upon PML/RARA-Negative AML Blasts Is Supported by All-Trans Retinoic Acid: In Vitro and in Vivo Evidence for Cooperation. Blood Cancer J..

[153] Li Y., He Y., Liang Z., Wang Y., Chen F., Djekidel M.N., Li G., Zhang X., Xiang S., Wang Z. (2018). Alterations of Specific Chromatin Conformation Affect ATRA-Induced Leukemia Cell Differentiation. Cell Death Dis..

[154] Trus M.R., Yang L., Suarez Saiz F., Bordeleau L., Jurisica I., Minden M.D. (2005). The Histone Deacetylase Inhibitor Valproic Acid Alters Sensitivity towards All Trans Retinoic Acid in Acute Myeloblastic Leukemia Cells. Leukemia.

[155] Costantini L., Molinari R., Farinon B., Merendino N. (2020). Retinoic Acids in the Treatment of Most Lethal Solid Cancers. J. Clin. Med..

[156] Mere Del Aguila E., Tang X.-H., Gudas L.J. (2022). Pancreatic Ductal Adenocarcinoma: New Insights into the Actions of Vitamin A. Oncol. Res. Treat..

[157] Parigiani M.A., Mandel M., Becker H. (2022).

[158] Carapuça E.F., Gemenetzidis E., Feig C., Bapiro T.E., Williams M.D., Wilson A.S., Delvecchio F.R., Arumugam P., Grose R.P., Lemoine N.R. (2016). Anti-Stromal Treatment Together with Chemotherapy Targets Multiple Signalling Pathways in Pancreatic Adenocarcinoma. J. Pathol..

[159] Wei S., Kozono S., Kats L., Nechama M., Li W., Guarnerio J., Luo M., You M.-H., Yao Y., Kondo A. (2015). Active Pin1 Is a Key Target of All-Trans Retinoic Acid in Acute Promyelocytic Leukemia and Breast Cancer. Nat. Med..

[160] Chronopoulos A., Robinson B., Sarper M., Cortes E., Auernheimer V., Lachowski D., Attwood S., García R., Ghassemi S., Fabry B. (2016). ATRA Mechanically Reprograms Pancreatic Stellate Cells to Suppress Matrix Remodelling and Inhibit Cancer Cell Invasion. Nat. Commun..

[161] Koikawa K., Kibe S., Suizu F., Sekino N., Kim N., Manz T.D., Pinch B.J., Akshinthala D., Verma A., Gaglia G. (2021). Targeting Pin1 Renders Pancreatic Cancer Eradicable by Synergizing with Immunochemotherapy. Cell.

[162] Kocher H.M., Basu B., Froeling F.E.M., Sarker D., Slater S., Carlin D., deSouza N.M., De Paepe K.N., Goulart M.R., Hughes C. (2020). Phase I Clinical Trial Repurposing All-Trans Retinoic Acid as a Stromal Targeting Agent for Pancreatic Cancer. Nat. Commun..

[163] Kocher H.M., Basu B., Froeling F.E.M., Sarker D., Slater S., Carlin D., Coetzee C., de Souza N., Goulart M., Hughes C. (2019). STAR-PAC: Phase I Clinical Trial Repurposing All Trans Retinoic Acid (ATRA) as Stromal Targeting Agent in a Novel Drug Combination for Pancreatic Cancer. Ann. Oncol..

[164] Phase IIb Randomised Clinical Trial Repurposing ATRA as a Stromal Targeting Agent in a Novel Drug Combination for Pancreatic Cancer. https://clinicaltrials.gov/ct2/show/NCT04241276.

[165] Luu T., Frankel P., Beumer J.H., Lim D., Cristea M., Appleman L.J., Lenz H.J., Gandara D.R., Kiesel B.F., Piekarz R.L. (2019). Phase I Trial of Belinostat in Combination with 13-Cis-Retinoic Acid in Advanced Solid Tumor Malignancies: A California Cancer Consortium NCI/CTEP Sponsored Trial. Cancer Chemother. Pharmacol..

[166] Tilsed C.M., Casey T.H., de Jong E., Bosco A., Zemek R.M., Salmons J., Wan G., Millward M.J., Nowak A.K., Lake R.A. (2022). Retinoic Acid Induces an IFN-Driven Inflammatory Tumour Microenvironment, Sensitizing to Immune Checkpoint Therapy. Front. Oncol..

[167] Treatment with Nivolumab and All-Trans Retinoic Acid for Patients with Refractory Pancreatic Cancer. https://www.clinicaltrials.gov/ct2/show/NCT05482451.

[168] Sahai V., Redig A.J., Collier K.A., Eckerdt F.D., Munshi H.G. (2016). Targeting BET Bromodomain Proteins in Solid Tumors. Oncotarget.

[169] Sahai V., Kumar K., Knab L.M., Chow C.R., Raza S.S., Bentrem D.J., Ebine K., Munshi H.G. (2014). BET Bromodomain Inhibitors Block Growth of Pancreatic Cancer Cells in Three-Dimensional Collagen. Mol. Cancer Ther..

[170] Jauset T., Massó-Vallés D., Martínez-Martín S., Beaulieu M.-E., Foradada L., Fiorentino F.P., Yokota J., Haendler B., Siegel S., Whitfield J.R. (2018). BET Inhibition Is an Effective Approach against KRAS-Driven PDAC and NSCLC. Oncotarget.

[171] Kumar K., DeCant B.T., Grippo P.J., Hwang R.F., Bentrem D.J., Ebine K., Munshi H.G. (2017). BET Inhibitors Block Pancreatic Stellate Cell Collagen I Production and Attenuate Fibrosis in Vivo. JCI Insight.

[172] Miller A.L., Garcia P.L., Fehling S.C., Gamblin T.L., Vance R.B., Council L.N., Chen D., Yang E.S., van Waardenburg R.C.A.M., Yoon K.J. (2021). The BET Inhibitor JQ1 Augments the Antitumor Efficacy of Gemcitabine in Preclinical Models of Pancreatic Cancer. Cancers.

[173] Garcia P.L., Miller A.L., Zeng L., van Waardenburg R.C.A.M., Yang E.S., Yoon K.J. (2022). The BET Inhibitor JQ1 Potentiates the Anticlonogenic Effect of Radiation in Pancreatic Cancer Cells. Front. Oncol..

[174] Xie F., Huang M., Lin X., Liu C., Liu Z., Meng F., Wang C., Huang Q. (2018). The BET Inhibitor I-BET762 Inhibits Pancreatic Ductal Adenocarcinoma Cell Proliferation and Enhances the Therapeutic Effect of Gemcitabine. Sci. Rep..

[175] Piha-Paul S.A., Sachdev J.C., Barve M., LoRusso P., Szmulewitz R., Patel S.P., Lara P.N., Chen X., Hu B., Freise K.J. (2019). First-in-Human Study of Mivebresib (ABBV-075), an Oral Pan-Inhibitor of Bromodomain and Extra Terminal Proteins, in Patients with Relapsed/Refractory Solid Tumors. Clin. Cancer Res..

[176] A Phase IB Trial with OTX015/MK-8628, a Small Molecule Inhibitor of the Bromodomain and Extra-Terminal (BET) Proteins, in Patients with Selected Advanced Solid Tumors. https://clinicaltrials.gov/ct2/show/NCT02259114.

[177] Mazur P.K., Herner A., Mello S.S., Wirth M., Hausmann S., Sánchez-Rivera F.J., Lofgren S.M., Kuschma T., Hahn S.A., Vangala D. (2015). Combined Inhibition of BET Family Proteins and Histone Deacetylases as a Potential Epigenetics-Based Therapy for Pancreatic Ductal Adenocarcinoma. Nat. Med..

[178] He S., Dong G., Li Y., Wu S., Wang W., Sheng C. (2020). Potent Dual BET/HDAC Inhibitors for Efficient Treatment of Pancreatic Cancer. Angew. Chem. Int. Ed..

[179] Miller A.L., Fehling S.C., Garcia P.L., Gamblin T.L., Council L.N., van Waardenburg R.C.A.M., Yang E.S., Bradner J.E., Yoon K.J. (2019). The BET Inhibitor JQ1 Attenuates Double-Strand Break Repair and Sensitizes Models of Pancreatic Ductal Adenocarcinoma to PARP Inhibitors. EBioMedicine.

[180] Phase Ib/II Study of ZEN003694 and Entinostat in Advanced and Refractory Solid Tumors and Lymphomas. https://clinicaltrials.gov/ct2/show/NCT05053971.

[181] Phase 1/2 Safety and Efficacy Study of NUV-868 as Monotherapy and in Combination with Olaparib or Enzalutamide in Adult Patients with Advanced Solid Tumors. https://clinicaltrials.gov/ct2/show/NCT05252390.

[182] Principe D.R., Xiong R., Li Y., Pham T.N.D., Kamath S.D., Dubrovskyi O., Ratia K., Huang F., Zhao J., Shen Z. (2022). XP-524 Is a Dual-BET/EP300 Inhibitor That Represses Oncogenic KRAS and Potentiates Immune Checkpoint Inhibition in Pancreatic Cancer. Proc. Natl. Acad. Sci. USA.

[183] Tu M., Klein L., Espinet E., Georgomanolis T., Wegwitz F., Li X., Urbach L., Danieli-Mackay A., Küffer S., Bojarczuk K. (2021). TNF-α-Producing Macrophages Determine Subtype Identity and Prognosis via AP1 Enhancer Reprogramming in Pancreatic Cancer. Nat. Cancer.

[184] Phase I/Ib Trial Evaluating the Safety and Efficacy of BET Inhibitor, ZEN003694 with PD-1 Inhibitor, Nivolumab with or without CTLA-4 Inhibitor, Ipilimumab in Solid Tumors. https://clinicaltrials.gov/ct2/show/NCT04840589.

[185] A Phase I/IIa Trial with BMS-986158, a Small Molecule Inhibitor of the Bromodomain and Extra-Terminal (BET) Proteins, as Monotherapy or in Combination with Nivolumab in Subjects with Selected Advanced Solid Tumors or Hematologic Malignancies. https://clinicaltrials.gov/ct2/show/NCT02419417.

[186] Li C., Wang Y., Gong Y., Zhang T., Huang J., Tan Z., Xue L. (2021). Finding an Easy Way to Harmonize: A Review of Advances in Clinical Research and Combination Strategies of EZH2 Inhibitors. Clin. Epigenetics.

[187] Qiu X., Wang W., Li B., Cheng B., Lin K., Bai J., Li H., Yang G. (2019). Targeting Ezh2 Could Overcome Docetaxel Resistance in Prostate Cancer Cells. BMC Cancer.

[188] Fillmore C.M., Xu C., Desai P.T., Berry J.M., Rowbotham S.P., Lin Y.-J., Zhang H., Marquez V.E., Hammerman P.S., Wong K.-K. (2015). EZH2 Inhibition Sensitizes BRG1 and EGFR Mutant Lung Tumours to TopoII Inhibitors. Nature.

[189] Karakashev S., Fukumoto T., Zhao B., Lin J., Wu S., Fatkhutdinov N., Park P.-H., Semenova G., Jean S., Cadungog M.G. (2020). EZH2 Inhibition Sensitizes CARM1-High, Homologous Recombination Proficient Ovarian Cancers to PARP Inhibition. Cancer Cell.

[190] Cai L., Wang Z., Liu D. (2016). Interference with Endogenous EZH2 Reverses the Chemotherapy Drug Resistance in Cervical Cancer Cells Partly by up-Regulating Dicer Expression. Tumour Biol..

[191] Avan A., Crea F., Paolicchi E., Funel N., Galvani E., Marquez V.E., Honeywell R.J., Danesi R., Peters G.J., Giovannetti E. (2012). Molecular Mechanisms Involved in the Synergistic Interaction of the EZH2 Inhibitor 3-Deazaneplanocin A with Gemcitabine in Pancreatic Cancer Cells. Mol. Cancer Ther..

[192] Guo Z., Sun Y., Liang L., Lu W., Luo B., Wu Z., Huo B., Hu Y., Huang P., Wu Q. (2022). Design and Synthesis of Dual EZH2/BRD4 Inhibitors to Target Solid Tumors. J. Med. Chem..

[193] Yap T.A., Winter J.N., Giulino-Roth L., Longley J., Lopez J., Michot J.-M., Leonard J.P., Ribrag V., McCabe M.T., Creasy C.L. (2019). Phase I Study of the Novel Enhancer of Zeste Homolog 2 (EZH2) Inhibitor GSK2816126 in Patients with Advanced Hematologic and Solid Tumors. Clin. Cancer Res..

[194] A Phase II Study of Tazemetostat in Solid Tumors Harboring an ARID1A Mutation. https://clinicaltrials.gov/ct2/show/NCT05023655.

[195] An Open-Label, Multicenter, Two-Part, Phase 1 Study to Characterize the Effects of a Moderate CYP3A Inhibitor on the Pharmacokinetics of Tazemetostat (EPZ-6438) (Part A), the Effects of Tazemetostat on the Pharmacokinetics of CYP2C8 and CYP2C19 Substrates, and the Effect of Increased Gastric PH on the Pharmacokinetics of Tazemetostat (Part B) in Subjects with B-Cell Lymphoma or Advanced Solid Tumors. https://clinicaltrials.gov/ct2/show/NCT03028103.

[196] Morschhauser F., Tilly H., Chaidos A., McKay P., Phillips T., Assouline S., Batlevi C.L., Campbell P., Ribrag V., Damaj G.L. (2020). Tazemetostat for patients with relapsed or refractory follicular lymphoma: An open-label, single-arm, multicentre, phase 2 trial. Lancet Oncol..

[197] An Open-Label Phase I/II Study of EZH2 Inhibitor SHR2554 in Combination with Anti-PD-L1/TGFβ Antibody SHR1701 in Patients with Advanced or Metastatic Solid Tumors and Relapsed/Refractory B-Cell Lymphomas. https://clinicaltrials.gov/ct2/show/NCT04407741.

[198] A Phase 1 Study of CPI-1205 with Ipilimumab in Patients with Advanced Solid Tumors Followed by a Phase 2 Basket Study of CPI-1205 with Ipilimumab in Selected Tumor Types Previously Treated with PD-1 or PD-L1 Inhibitors. https://clinicaltrials.gov/ct2/show/NCT03525795.

[199] Combining Epigenetic and Immune Therapy to Beat Cancer. CAIRE Study. https://clinicaltrials.gov/ct2/show/NCT04705818.

[200] Zhou L., Mudianto T., Ma X., Riley R., Uppaluri R. (2020). Targeting EZH2 Enhances Antigen Presentation, Antitumor Immunity, and Circumvents Anti-PD-1 Resistance in Head and Neck Cancer. Clin. Cancer Res..

[201] Goswami S., Apostolou I., Zhang J., Skepner J., Anandhan S., Zhang X., Xiong L., Trojer P., Aparicio A., Subudhi S.K. (2018). Modulation of EZH2 Expression in T Cells Improves Efficacy of Anti-CTLA-4 Therapy. J. Clin. Investig..

[202] Jin N., George T.L., Otterson G.A., Verschraegen C., Wen H., Carbone D., Herman J., Bertino E.M., He K. (2021). Advances in Epigenetic Therapeutics with Focus on Solid Tumors. Clin. Epigenetics.

[203] Neureiter D., Zopf S., Leu T., Dietze O., Hauser-Kronberger C., Hahn E.G., Herold C., Ocker M. (2007). Apoptosis, Proliferation and Differentiation Patterns Are Influenced by Zebularine and SAHA in Pancreatic Cancer Models. Scand. J. Gastroenterol..

[204] Raghavan S., Winter P.S., Navia A.W., Williams H.L., DenAdel A., Lowder K.E., Galvez-Reyes J., Kalekar R.L., Mulugeta N., Kapner K.S. (2021). Microenvironment Drives Cell State, Plasticity, and Drug Response in Pancreatic Cancer. Cell.

[205] Chan-Seng-Yue M., Kim J.C., Wilson G.W., Ng K., Figueroa E.F., O’Kane G.M., Connor A.A., Denroche R.E., Grant R.C., McLeod J. (2020). Transcription Phenotypes of Pancreatic Cancer Are Driven by Genomic Events during Tumor Evolution. Nat. Genet..

[206] Niemöller C., Wehrle J., Riba J., Claus R., Renz N., Rhein J., Bleul S., Stosch J.M., Duyster J., Plass C. (2021). Bisulfite-Free Epigenomics and Genomics of Single Cells through Methylation-Sensitive Restriction. Commun. Biol..

[207] Gupta S., Pramanik D., Mukherjee R., Campbell N.R., Elumalai S., de Wilde R.F., Hong S.-M., Goggins M.G., De Jesus-Acosta A., Laheru D. (2012). Molecular Determinants of Retinoic Acid Sensitivity in Pancreatic Cancer. Clin. Cancer Res..

[208] Azad N., Zahnow C.A., Rudin C.M., Baylin S.B. (2013). The Future of Epigenetic Therapy in Solid Tumours—Lessons from the Past. Nat. Rev. Clin. Oncol..

[209] Majchrzak-Celińska A., Warych A., Szoszkiewicz M. (2021). Novel Approaches to Epigenetic Therapies: From Drug Combinations to Epigenetic Editing. Genes.

[210] Lechner S., Malgapo M.I.P., Grätz C., Steimbach R.R., Baron A., Rüther P., Nadal S., Stumpf C., Loos C., Ku X. (2022). Target Deconvolution of HDAC Pharmacopoeia Reveals MBLAC2 as Common off-Target. Nat. Chem. Biol..

[211] Gravina G.L., Festuccia C., Marampon F., Popov V.M., Pestell R.G., Zani B.M., Tombolini V. (2010). Biological Rationale for the Use of DNA Methyltransferase Inhibitors as New Strategy for Modulation of Tumor Response to Chemotherapy and Radiation. Mol. Cancer.

[212] Bondarev A.D., Attwood M.M., Jonsson J., Chubarev V.N., Tarasov V.V., Schiöth H.B. (2021). Recent Developments of HDAC Inhibitors: Emerging Indications and Novel Molecules. Br. J. Clin. Pharmacol..

[213] Doroshow D.B., Eder J.P., LoRusso P.M. (2017). BET Inhibitors: A Novel Epigenetic Approach. Ann. Oncol..

[214] Pappalardi M.B., Keenan K., Cockerill M., Kellner W.A., Stowell A., Sherk C., Wong K., Pathuri S., Briand J., Steidel M. (2021). Discovery of a First-in-Class Reversible DNMT1-Selective Inhibitor with Improved Tolerability and Efficacy in Acute Myeloid Leukemia. Nat. Cancer.

[215] Porcu P., Haverkos B., Brem E., Vallurupalli A., Feldman T., Alpdogan O., Brammer J.E., Bryan L.J., Barta S.K., Obrzut S. (2019). A Phase Ib/II Study of Oral Nanatinostat (N) and Valganciclovir (VG) in Subjects with Epstein-Barr Virus (EBV)-Associated Lymphomas. J. Clin. Oncol..

[216] Yee A.J., Bensinger W.I., Supko J.G., Voorhees P.M., Berdeja J.G., Richardson P.G., Libby E.N., Wallace E.E., Birrer N.E., Burke J.N. (2016). Ricolinostat plus Lenalidomide, and Dexamethasone in Relapsed or Refractory Multiple Myeloma: A Multicentre Phase 1b Trial. Lancet Oncol..

